# Novel polyadenylylation-dependent neutralization mechanism of the HEPN/MNT toxin/antitoxin system

**DOI:** 10.1093/nar/gkaa855

**Published:** 2020-10-12

**Authors:** Jianyun Yao, Xiangkai Zhen, Kaihao Tang, Tianlang Liu, Xiaolong Xu, Zhe Chen, Yunxue Guo, Xiaoxiao Liu, Thomas K Wood, Songying Ouyang, Xiaoxue Wang

**Affiliations:** Key Laboratory of Tropical Marine Bio-resources and Ecology, Guangdong Key Laboratory of Marine Materia Medica, Innovation Academy of South China Sea Ecology and Environmental Engineering, South China Sea Institute of Oceanology, Chinese Academy of Sciences, 164 West Xingang Road, Guangzhou 510301, China; Southern Marine Science and Engineering Guangdong Laboratory (Guangzhou), No.1119, Haibin Road, Nansha District, Guangzhou 511458, China; Provincial University Key Laboratory of Cellular Stress Response and Metabolic Regulation, The Key Laboratory of Innate Immune Biology of Fujian Province, Biomedical Research Center of South China, Key Laboratory of OptoElectronic Science and Technology for Medicine of the Ministry of Education, College of Life Sciences, Fujian Normal University, Fuzhou, China; Key Laboratory of Tropical Marine Bio-resources and Ecology, Guangdong Key Laboratory of Marine Materia Medica, Innovation Academy of South China Sea Ecology and Environmental Engineering, South China Sea Institute of Oceanology, Chinese Academy of Sciences, 164 West Xingang Road, Guangzhou 510301, China; Southern Marine Science and Engineering Guangdong Laboratory (Guangzhou), No.1119, Haibin Road, Nansha District, Guangzhou 511458, China; Key Laboratory of Tropical Marine Bio-resources and Ecology, Guangdong Key Laboratory of Marine Materia Medica, Innovation Academy of South China Sea Ecology and Environmental Engineering, South China Sea Institute of Oceanology, Chinese Academy of Sciences, 164 West Xingang Road, Guangzhou 510301, China; Southern Marine Science and Engineering Guangdong Laboratory (Guangzhou), No.1119, Haibin Road, Nansha District, Guangzhou 511458, China; University of Chinese Academy of Sciences, Beijing, China; Provincial University Key Laboratory of Cellular Stress Response and Metabolic Regulation, The Key Laboratory of Innate Immune Biology of Fujian Province, Biomedical Research Center of South China, Key Laboratory of OptoElectronic Science and Technology for Medicine of the Ministry of Education, College of Life Sciences, Fujian Normal University, Fuzhou, China; Key Laboratory of Tropical Marine Bio-resources and Ecology, Guangdong Key Laboratory of Marine Materia Medica, Innovation Academy of South China Sea Ecology and Environmental Engineering, South China Sea Institute of Oceanology, Chinese Academy of Sciences, 164 West Xingang Road, Guangzhou 510301, China; Southern Marine Science and Engineering Guangdong Laboratory (Guangzhou), No.1119, Haibin Road, Nansha District, Guangzhou 511458, China; University of Chinese Academy of Sciences, Beijing, China; Key Laboratory of Tropical Marine Bio-resources and Ecology, Guangdong Key Laboratory of Marine Materia Medica, Innovation Academy of South China Sea Ecology and Environmental Engineering, South China Sea Institute of Oceanology, Chinese Academy of Sciences, 164 West Xingang Road, Guangzhou 510301, China; Southern Marine Science and Engineering Guangdong Laboratory (Guangzhou), No.1119, Haibin Road, Nansha District, Guangzhou 511458, China; Key Laboratory of Tropical Marine Bio-resources and Ecology, Guangdong Key Laboratory of Marine Materia Medica, Innovation Academy of South China Sea Ecology and Environmental Engineering, South China Sea Institute of Oceanology, Chinese Academy of Sciences, 164 West Xingang Road, Guangzhou 510301, China; Southern Marine Science and Engineering Guangdong Laboratory (Guangzhou), No.1119, Haibin Road, Nansha District, Guangzhou 511458, China; Department of Chemical Engineering, Pennsylvania State University, University Park, Pennsylvania 16802-4400, USA; Provincial University Key Laboratory of Cellular Stress Response and Metabolic Regulation, The Key Laboratory of Innate Immune Biology of Fujian Province, Biomedical Research Center of South China, Key Laboratory of OptoElectronic Science and Technology for Medicine of the Ministry of Education, College of Life Sciences, Fujian Normal University, Fuzhou, China; Key Laboratory of Tropical Marine Bio-resources and Ecology, Guangdong Key Laboratory of Marine Materia Medica, Innovation Academy of South China Sea Ecology and Environmental Engineering, South China Sea Institute of Oceanology, Chinese Academy of Sciences, 164 West Xingang Road, Guangzhou 510301, China; Southern Marine Science and Engineering Guangdong Laboratory (Guangzhou), No.1119, Haibin Road, Nansha District, Guangzhou 511458, China; University of Chinese Academy of Sciences, Beijing, China

## Abstract

The two-gene module HEPN/MNT is predicted to be the most abundant toxin/antitoxin (TA) system in prokaryotes. However, its physiological function and neutralization mechanism remains obscure. Here, we discovered that the MntA antitoxin (MNT-domain protein) acts as an adenylyltransferase and chemically modifies the HepT toxin (HEPN-domain protein) to block its toxicity as an RNase. Biochemical and structural studies revealed that MntA mediates the transfer of three AMPs to a tyrosine residue next to the RNase domain of HepT in *Shewanella oneidensis*. Furthermore, *in vitro* enzymatic assays showed that the three AMPs are transferred to HepT by MntA consecutively with ATP serving as the substrate, and this polyadenylylation is crucial for reducing HepT toxicity. Additionally, the GSX_10_DXD motif, which is conserved among MntA proteins, is the key active motif for polyadenylylating and neutralizing HepT. Thus, HepT/MntA represents a new type of TA system, and the polyadenylylation-dependent TA neutralization mechanism is prevalent in bacteria and archaea.

## INTRODUCTION

Toxin-antitoxin (TA) systems are small genetic elements found on plasmids and in the chromosomes of many bacteria and archaea. They serve to either reduce metabolism during stress (1,2) or to inhibit phage infection (3–5). Currently, at least six different types of TA systems have been identified based on the nature of antitoxins (protein or RNA) as well as the way an antitoxin interacts with a toxin to block its toxicity. In type I and type III TA systems, the antitoxin is RNA, and the antitoxin blocks the toxicity of the toxin either by RNA–RNA interactions or RNA–protein interactions. For the rest of the TA systems, the antitoxins are proteins, and the way they block the toxicity of the cognate toxin varies. In type II systems, the antitoxin binds to the toxin, and the neutralization of the toxin depends on direct protein-protein interactions with the toxin. In type IV systems, the antitoxin and the toxin do not interact but act on the same target (6,7). In type V systems, the antitoxin specifically cleaves the toxin mRNA to inhibit toxin production (8). In type VI systems, when the TA complex is formed, the antitoxin facilitates the degradation of the toxin (9). Recently, several novel TA systems have been reported in which the antitoxin functions as an enzyme: Hha/TomB (antitoxin oxidizes Cys of the toxin) (10), ToxSAS/antiToxSAS (antitoxin degrades the product of the toxin, (p)ppGpp) (11), and TglT/TakA (antitoxin phosphorylates Ser of the toxin) (12), expanding the ways antitoxins neutralize their toxins.

The two-gene module encoding the HEPN (higher eukaryotes and prokaryotes nucleotide-binding) domain and cognate MNT (minimal nucleotidyltransferase) domain has been predicted to represent one of the most abundant TA systems in archaea and bacteria (13). The MNT domain-containing protein appeared to be the only active enzyme in the putative TA system, so it was predicted to be the toxin (14). Unexpectedly, a genome-wide screen for TA modules using shotgun cloning led to the characterization of the HEPN domain-containing protein HhalT as the toxin and the neighboring MNT domain-containing protein HhalA as the antitoxin in the halophilic bacterium *Halorhodospira halophila* SL1 (15). Our subsequent work demonstrated that the HEPN protein with a RX_4–6_H motif is the toxin which cleaves mRNA *in vitro* in the HEPN/MNT (SO_3166/SO_3165) TA pair in the model psychrotrophic bacterium *Shewanella oneidensis* (16). Furthermore, antitoxin SO_3165 binds to toxin SO_3166 at a rare 2:6 ratio to form a hetero-octamer, and this binding is important for the antitoxin to block the toxicity of the toxin (17). These results demonstrate that the HEPN/MNT module constitutes a *bona fide* TA system and resembles a type II TA system as both components are proteins that interact with each other. Noticeably, unlike other typical type II antitoxins, MNTs adopt a minimal domain of the polβ nucleotidyltransferase superfamily instead of a DNA-binding domain (18). However, these MNTs seem to contain only the catalytic domain but lack the region for substrate recognition usually present in known nucleotidyltransferases due to the size limitations (∼80–150 aa) (19). Thus, whether these antitoxins retain nucleotidyltransferase activity is unclear.

Here, by studying the representative HEPN/MNT TA pairs in Proteobacteria and Euryarchaeota, we discovered that the MNT antitoxin adenylylates the cognate HEPN toxin to inactive HEPN. A distinctive feature of the HepT (HEPN toxin)/MntA (MNT antitoxin) TA pair is a conserved RX_4_HXY motif for HepT and a conserved GSX_10_DXD motif for MntA. Specifically, structural and biochemical studies of the HepT/MntA TA pair (SO_3166/SO_3165) in *S. oneidensis* show that the GSX_10_DXD motif of antitoxin MntA is responsible for transferring three AMPs to HepT and that Y104 in RX_4_HXY in HepT is the site that receives three AMPs from MntA. Amino acid substitution experiments demonstrate that mutating the signature motif in MntA not only abrogates the adenylyltransferase activity of MntA but also reduces the ability of MntA to neutralize HepT toxicity. *in vitro* enzymatic assays showed that the three AMPs are transferred to HepT by MntA consecutively using ATP as the substrate. Structural analysis of the HepT/MntA TA complex containing modified or un-modified HepT indicates that the three AMPs are located near the active RNase domain of HepT and can interfere with the substrate binding sites. Critically, the HepT/MntA TA pair with signature motifs represents a new type of TA system that is prevalent in both bacteria and archaea. Likewise, we show the HepT toxin homolog in the hyperthermophilic archaeon *Thermococcus cleftensis* is also adenylylated by the cognate MntA homolog. The prevalence of these HEPN/MNT modules in bacteria and archaea suggest that these TA systems may be involved in the environmental adaption to extreme habitats.

## MATERIALS AND METHODS

### Bacterial strains, plasmids and growth conditions

The strains and plasmids used in this study are listed in Supplementary Table S1. Experiments were conducted at 30°C for *S. oneidensis* and at 37°C for *Escherichia coli* in Luria-Bertani (LB) medium. 2,6-diamino-pimelic acid was added to *E. coli* WM3064 at a final concentration of 0.3 mM. Chloramphenicol (30 μg/ml) and kanamycin (50 μg/ml) were added to cells harboring plasmids with the indicated resistance genes listed in Supplementary Table S1. When required, 0.1–1 mM IPTG (isopropyl-β-d-thiogalactopyranoside) was added as an inducer.

### Mutagenesis

The mutations in *hepT* or *mntA* were introduced by using a modified two-step PCR method via two inside primers and two outside primers (20). PCR products using one of the inside primers containing the mutated sites was combined with one of the outside primers covering the whole gene or operon to generate a template for the second round of PCR, and the pair of outside primers were used for the second-round PCR to fuse the two PCR fragments from the first-round PCR (primers listed in Supplementary Table S2). The two insider primers both have the mutations and they are of opposite directions. All the mutants were confirmed by DNA sequencing.

### Protein purification and western blot assays

*Escherichia coli* BL21 (DE3) strains carrying pET28b-based plasmids were grown in LB supplemented with kanamycin and induced with 0.2 mM IPTG for 12 h at 25°C. Specifically, in order to purify toxin HepT and HepT/MntA variants that are toxic, stationary cells (OD_600_ ∼ 1.0) were induced with 0.5 mM IPTG for only 2 h at 37°C (longer induction periods and IPTG addition at inoculation resulted in too much toxicity). Cells expressing the protein of interest were collected, and the His-tagged proteins were purified following the method described in a previous study (21). For the western blot assay, protein samples were transferred to a PVDF membrane (Millipore, Bedford, MA, USA), and performed with primary antibodies raised against a His-tag (Cell Signaling Technology, Danvers, MA, USA) and horseradish peroxidase-conjugated goat anti-mouse secondary antibodies (Bio-Rad, Richmond, CA, USA).

### Phosphodiesterase (PDE) cleavage assay

To conduct the PDE cleavage assay, the HepT/MntA TA complex and mutants (HepT^Y104A^/MntA, HepT/MntA^G27A, S28T^) were purified as described above. The reaction mixture for the cleavage assay contained 20 mM Tris–HCl (pH 8.0) and 10 μg of purified protein and was incubated with PDE (2 U μl^−1^) at 37°C for 5–40 min. The small molecule products of these reaction mixes were collected using a 3K membrane for further identification.

### Liquid chromatography–mass spectrometry analysis (LC–MS)

The products collected in the PDE treatment assays were analyzed using LC–MS. Electrospray ionization mass spectrometry (ESI-MS) was performed in positive/negative mode using an integrated HPLC/ESI-MS system (1260 Infinity, Agilent Technologies/amaZon SL, Bruker Technologies) equipped with a Luna 5u C18 column (100A, 250 × 4.60 mm, 5 μm). Elution was performed with a linear gradient of solvents A (0.1% formic acid (FA) in water, pH 7.0) and B (acetonitrile) at a flow of 1.0 ml/min as follows: 0–5 min, 1–5% B; 5–10 min, 5–10% B; 10–15 min, 10–5% B; 15–20 min, 5–1%. The ionization capillary voltage was set to 4500 V, and the fragmentor was set to 150 V.

### HPLC/Q-TOF-MS analysis

Identification of the molecular mass was carried out by MS detection using a quadrupole time-of-flight instrument coupled to an HPLC (Agilent Technologies). The elution gradient employed for the separation of proteins in each fraction was as follows: 0–2 min, 2% B; 2–8 min, 2–50% B; 8–12 min, 50–98% B; 12–16 min, 98% B; 16–17.1 min, 98–2% B; 17.1–20 min, 2% B. Mobile phases A and B consisted of water and acetonitrile, with both phases containing 0.1% (v/v) FA. Protein samples were separated by HPLC, and an MS-range from *m*/*z* 600 to 2000 was employed. The deconvolution analysis was displayed by the software Deconvolute (MS): protein (Agilent) using the deconvolution algorithm Maximum Entropy method.

### Crystallization and data collection

The purified HepT and MntA proteins were concentrated in 0.5 ml and loaded onto a Superdex 200 column (GE Healthcare) equilibrated with 20 mM Tris (pH 8.0) and 150 mM NaCl. Fractions containing the target proteins were concentrated, and crystallization was performed using the sitting drop vapor diffusion method at 16°C. Crystallization drops containing 0.5 μl of the protein solution were mixed with 0.5 μl of reservoir solution. The protein concentration of HepT/MntA used for crystallization was ∼20 mg/ml. Diffraction quality crystals of the HepT/MntA complex were grown in the presence of 0.1 M Tris–HCl (pH 7.8–8.2), 0.2 M potassium sodium tartrate, and 12–20% (v/v) PEG 3350. Crystals were harvested with 20% (v/v) glycol as a cryoprotectant before flash freezing them in liquid nitrogen. HepT/MntA^D39E, D41E^ and HepT^Y104A^/MntA were purified as the same procedure and the crystals were obtained in the 0.1 M HEPES pH 7.5 0.15 M, 0.2 M lithium sulfate monohydrate and 20% PEG3350. To obtain the complex of HepT^Y104A^/MntA bound with the substrate, AMP-PNP was used for co-crystallization with HepT^Y104A^/MntA at a 10:1 molar ratio at 4°C for 30 min, crystals were grown using the same conditions. X-ray diffraction data were collected on BL-17U1 at Shanghai Synchrotron Radiation Facility (SSRF). The diffraction images were processed with the HKL2000 program (22).

### Structure determination and refinement

The crystal structures of HepT/MntA, HepT/MntA^D39E,D41E^, HepT^Y104A^/MntA and HepT^Y104A^/MntA-AMP-PNP were solved via the molecular replacement (MR) method with Phaser using HepT/MntA (PDB code 5YEP) as the search template. Model building and structure refinement were performed manually in Coot (23) and automatically in PHENIX (24). The stereochemical analysis of the final models was performed by MOLPROBITY (25). Data collection and refinement statistics are presented in Table 1.

**Table 1.** X-ray data collection and refinement statistics of TA complex

Dataset	HepT/MntA	HepT^Y104A^/MntA	HepT/ MntA^D39E, D41E^	HepT^Y104A^/MntA-AMP-PNP
**Data collection**				
Wavelength (Å)	0.9792	0.9792	0.9792	0.9792
Space group	*P* 2_1_ 2_1_ 2	*P* 1 2_1_ 1	*P* 1 2_1_ 1	*P* 1 2_1_ 1
Cell dimensions				
*a*, *b*, *c* (Å)	56.56, 225.70, 52.74	54.12, 100.85, 131.82	54.51, 100.68, 132.51	54.51 100.68 132.51
α, β, γ (°)	90.00, 90.00, 90.00	90.00, 97.08, 90.00	90.00, 96.44, 90.00	90.00, 96.44, 90.00
Resolution range (Å)	112.8–3.08 (3.16–3.08)	100.85–2.61 (2.67–2.6)	100.10–2.35 (2.41–2.35)	100.68–2.77 (2.84–2.77)
Rmerge	0.196 (1.369)	0.069 (0.875)	0.059 (1.079)	0.082 (1.079)
CC1/2	0.997 (0.825)	0.998 (0.775)	0.999 (0.744)	0.999 (0.999)
I/σ(I)	10.2 (2.3)	14.3 (2.4)	16.6 (2.2)	15.9 (2.2)
Completeness (%)	99.25 (98.75)	98.7 (98.6)	99.74 (99.86)	99.9 (99.9)
Multiplicity	12.5 (13.2)	6.8 (6.9)	6.8 (7.2)	6.8 (7.1)
**Refinement**				
Resolution (Å)	112.8–3.08 (3.19–3.08)	39.19–2.61 (2.70–2.61)	53.91–2.35 (2.434–2.35)	32.37–2.77 (2.869–2.77)
*R*_work_ (%)	22.12 (35.66)	21.44 (33.48)	19.20 (31.28)	0.2191 (0.3185)
*R*_free_ (%)	27.70 (43.05)	24.95 (39.91)	23.56 (38.02)	0.2530 (0.3542)
Ramachandran plot (%)				
Favored region	99.01	97.00	95.02	95.41
Allowed region	0.80	2.90	3.81	3.32
Outliers region	0.70	0.60	0.60	0.88

### *In vitro* enzymatic assays

The *in vitro* adenylylation assay using purified unmodified HepT and MntA was modified based on the *in vitro* assay of Truttmann *et al.* (26). The unmodified HepT and MntA proteins are very similar in size (133 aa and 139 aa, respectively), making it difficult to distinguish the reaction products. Therefore, 24 extra aa (including the 6× His tag) were fused to the N terminus of MntA, a strategy we previously used that has potentially minimal effect on the activity of MntA (27). Purified MntA (10 μg) was added to 40 μl of reaction buffer (containing 20 mM Tris–HCl, 10 mM MgCl_2_ and 5 mM DTT) supplemented with 0 to 12 μM ATP and incubated at room temperature for 30 min. Unmodified HepT (20 μg) was then added to the reaction and incubated at 37°C for another 60 min. The reaction was stopped by adding SDS-PAGE loading buffer and the samples were analyzed by tricine-SDS-PAGE. To capture the intermediate products, a reaction with 3 μM ATP serving as the substrate was stopped by adding EDTA, and the products were analyzed by HPLC/Q-TOF-MS as described above.

For the *in vitro* RNase cleavage assay, *ompA* mRNA was synthetized, and the assay was performed as previously described using various of concentrations of HepT (0–160 nM) (17). The reaction mixture was incubated at 37°C for 30 min. To obtain the polyadenylylated HepT protein, the lysate from cells coexpressing *hepT* and *mntA* was shaken vigorously after incubating with Ni-NTA agarose, and polyadenylylated HepT with purity >90%, was obtained after a second elution (Supplementary Figure S1).

### The bacterial two-hybrid (BACTH) assay

For the BACTH assays, the coding region of *hepT* was inserted into pKT25, and the coding region of *mntA* was inserted into pUT18C. The recombinant plasmids were co-transformed into *E. coli* BTH101(cya-99) competent cells and the quantitative analyses of the BACTH assay was performed as previously described (21).

### Analysis of the distribution of HepT/MntA

HepT belongs to the Pfam family PF01934 (protein of unknown function with DUF86 belonging to clan CL0291) and MntA belongs to PF01909 (nucleotidyltransferase domain belonging to clan CL0260). Therefore, proteins belonging to CL0291 or CL0260 in 86 934 genomes (Supplementary Table S3) were retrieved using the function profile tool in the IMG/M system (Pfam v30) (28). Next, FIMO (29) was used to scan for proteins belonging to CL0291 that contain the RX_4_HXY motif or CL0260 that contain GSX_10_DXD. The initial hits were further curated by checking whether these two motifs were encoded by neighboring genes. This generated a total of 15 977 pairs, belonging to PF01934/PF01909 or PF08780/PF01909. This set was defined as Class I HEPN/MNT TA pairs. The Class II HEPN/MNT TA pairs contain the HEPN toxins from PF05168 but they do not contain the RX_4_HXY motif. In order to extensively evaluate the distribution of the HEPN/MNT pairs, both Class I and Class II proteins were recovered from the IMG/M database by the function profile tool (Supplementary Table S4). The motif of the each of two classes was generated by MEME (30).

## RESULTS

### Antitoxin MntA adenylylates toxin HepT in *S. oneidensis*

**Figure 1.**
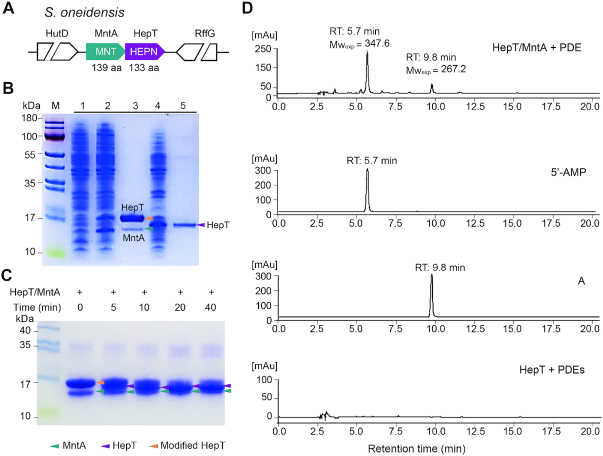
MntA-mediated adenylylation of HepT in *S. oneidensis*. (**A**) Genomic location of the HepT (SO_3166)/MntA (SO_3165) pair and neighboring genes in *S. oneidensis*. (**B**) *E. coli* BL21 containing pET28b served as a negative control (lane 1). His-tagged HepT and untagged MntA were induced from pET28b*-mntA-hepT-*His (lane 2) and further purified (lane 3). His-tagged HepT was induced from pET28b-*hepT*-His (lane 4) and further purified (lane 5). (**C**) Digestion of the HepT/MntA complex over time by PDE. (**D**) Characterization of the products cleaved by PDE using HPLC-MS. Identified masses (Mw_exp_, in Daltons) and compounds are presented above the chromatogram. 5′-AMP and A were included as the standards. The peaks were collected at 260 nm, and mAu is milli absorbance units.

Previously, we showed that SO_3165 (here, we suggest the name MntA) and SO_3166 (we suggest the name HepT) of the HEPN/MNT module in *S. oneidensis*, form a TA pair (16) (Figure 1A). However, during purification of the toxin protein HepT, we found that the mobility of HepT varied with and without MntA during SDS-PAGE fractionation. The vector pET28b-*mntA-hepT*-His_6_ was constructed to co-express a C-terminal His_6_-tagged HepT and untagged MntA, while pET28b-*hepT*-His_6_ was constructed to only express His_6_-tagged HepT. To exclude any influence of the resident HEPN/MNT module on our experiments, we typically used *E. coli* strains that did not have endogenous HEPN/MNT modules. Noticeably, HepT migrated slower when co-produced with MntA compared to when it was produced alone (Figure 1B). The molecular mass of the unmodified His_6_-tagged HepT was 16.14 kDa (Figure 1B and Supplementary Table S5), and the size of the unmodified and untagged MntA, which was pulled down due to its interaction with HepT, was 15.62 kDa (Figure 1B, lane 3). The presence of MntA led to the production of HepT with a molecular mass shift of ∼1 kDa higher than expected, suggesting that HepT might be modified in the presence of MntA. Thus, we also reasoned that this modification may involve the addition of a more complex chemical group than phosphate or acetyl, which are usually not detected by SDS-PAGE analysis.

MntA is a small protein of 139 aa and displays a minimal nucleotidyltransferase domain previously found by Aravind and Koonin (31). Since nucleotidyltransferase catalyzes the transfer of a nucleotide to an acceptor hydroxyl group, we hypothesized that MntA might transfer nucleotides to the side chains of HepT with hydroxyl groups. When a nucleotidyltransferase acts on its amino acid substrate, a phosphodiester bond is formed that is susceptible to cleavage by PDEs. To test this hypothesis, we purified the *S. oneidensis* HepT/MntA TA complex and treated the purified TA complex with snake venom PDE. As expected, HepT shifted back to the expected and unmodified size when treated with PDE (Figure 1C), suggesting that PDE cleaved the modified moiety from HepT. Next, we sought to identify the modified nucleotide in the cleaved products. HPLC/MS analysis of the cleaved products revealed that the major product had an MW of 347.6 Da with a retention time of 5.7 min, which was the same as the control adenosine 5′ monophosphate (5′-AMP) (Figure 1D and Supplementary Figure S2). Together, these results show that toxin HepT is adenylylated by antitoxin MntA.

### Key motif for adenylylation in HepT/MntA TA pair

**Figure 2.**
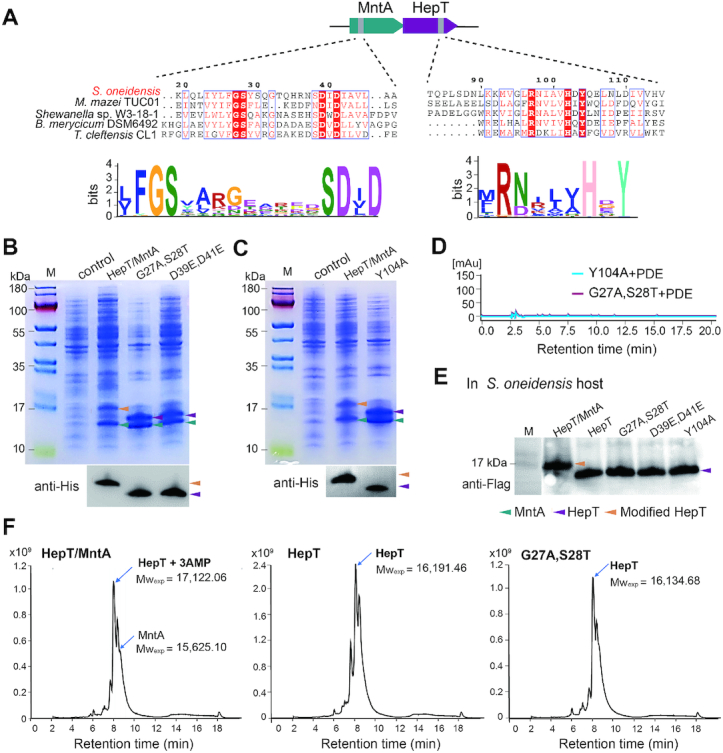
Key residues in HepT and MntA for receiving and transferring AMPs, respectively. (**A**) Protein sequences of HepT and MntA homologs were aligned in Supplementary Figure S3. A total of 15,094 sequences of MntA and HepT homologs were used for motif scanning, and the sequence profiles of the conserved motifs are shown. (**B**) His-tagged HepT was produced with untagged MntA (HepT/MntA), MntA^G27S, S28T^ (G27S, S28T) or MntA^D39E, D41E^ (D39E, D41E) from pET28b-based plasmids. (**C**) His-tagged HepT or HepT^Y104A^ (Y104A) were produced with untagged MntA from pET28b-based plasmids. The empty pET28b vector was used as the control, and western blot (lower panel) was performed using monoclonal anti-His tag antibodies in B and C. The arrowheads shown in panels B and C are the same as in E. (**D**) The products of the HepT/MntA^G27A, S28T^ and HepT ^Y104A^/MntA complex cleaved by PDE were analyzed using HPLC-MS. The peaks were collected at 260 nm. (**E**) *S. oneidensis* cells harboring pHGE-based vectors were induced with 0.5 mM IPTG for 2 h. Western blot was performed using monoclonal anti-Flag antibodies. (**F**) The experimentally identified MWs of TA complex analyzed by HPLC/Q-TOF-MS. The theoretical MWs of these proteins are listed in Supplementary Table S5. At least three independent protein purifications were performed for B and C, and only one representative image is shown here.

Since many proteins with nucleotidyltransferase domains are adjacent to proteins with HEPN domains, we investigated the conserved motif of MntA along with a diverse set of other HEPN/MNT-associated proteins. MntA and HepT belong to the PF01909 and PF01934 families (Pfam models: NTP_transf_2 and DUF86), respectively. Sequence analysis revealed that these MNT proteins included in the PF01909/PF01934 pairs have a highly conserved GSX_10_DXD (where X is any amino acid) motif (Figure 2A and Supplementary Figure S3). We thus performed site mutagenesis on the GSX_10_DXD motif of *S. oneidensis* MntA to explore whether it plays a role in the modification of HepT in the *E. coli* host. Substituting GS with AT in the GSX_10_DXD motif of MntA eliminated the modification of HepT as shown by SDS-PAGE fractionation and western blotting (Figure 2B and Supplementary Figure S4). Similarly, substituting the Asp of GSX_10_DXD with Glu (GSX_10_EXE) in MntA also eliminated the modification of HepT in the mutated TA complex (HepT/MntA^D39E, D41E^) (Figure 2B). These results demonstrate that this motif is critical for the modification of HepT.

Next, we identified the adenylylated site in HepT. Adenylylation involves the formation of a phosphodiester bond between the hydroxyl group of the side chain of the amino acid and the phosphate group of AMP. The covalent addition of an AMP moiety to Ser, Thr or Tyr has been previously reported (32). More importantly, we also noticed that the HEPNs neighboring the MNTs in the PFAM database have a signature motif of RX_4_HXY (where X is any amino acid) (Figure 2A). In addition to the reported active RNase RX_4_H motif of HEPNs, we found that Y104 in the RX_4_HXY motif is highly conserved, which has been also previously reported (13). To further check whether the Y104 residue in HepT is adenylylated *in vivo*, we performed site-directed mutagenesis on *hepT* of *S. oneidensis* at the codon for Y104 to yield the substitution Y104A. We found that the mobility of the substituted HepT toxin (HepT^Y104A^) produced by pET28b-*mntA-hepT*^Y104A^-His_6_ was the same as that produced by pET28b-*hepT*-His_6_ during SDS-PAGE fractionation and western blot analysis in an *E. coli* host (Figure 2C and Supplementary Figure S4). The results clearly show that substitution of the Y104 residue of HepT prevents adenylylation by MntA. As expected, no AMP moiety in the HepT^Y104A^/MntA complex or the HepT/MntA^G27A, S28T^ complex was found after PDE treatment by LC-MS analysis (Figure 2D). To investigate whether MntA can modify HepT in its original host *S. oneidensis*, a FLAG-tag was fused to the C-terminus of the toxin, and western blot was performed to check the status of HepT in the following constructs: HepT/MntA, HepT, HepT/MntA^G27A,S28T^, HepT/MntA^D39E,D41E^ and HepT^Y104A^/MntA. In agreement with the results in *E. coli*, HepT toxin is modified by native MntA in *S. oneidensis*, and substitutions in the MntA adenylylation motif eliminate HepT modification in *S. oneidensis* (Figure 2E and Supplementary Figure S4). The molecular masses of the native HepT/MntA TA complex, HepT alone, and the mutated HepT/MntA^G27A, S28T^ TA complex were analyzed by HPLC/Q-TOF-MS. The results revealed that the MW of adenylylated HepT increased by 987.45 Da compared to unmodified HepT, and this change in MW is equal to three AMPs (one AMP is 329 Da) (Figure 2F and Supplementary Figure S5). This result agrees with the SDS-PAGE analysis, where the MW of adenylylated HepT increase by ∼1 kDa (Figure 1B). Collectively, these results demonstrate that MntA modifies HepT by transferring three AMPs to HepT.

### Structural analysis revealed that Y104 of HepT is polyadenylylated

**Figure 3.**
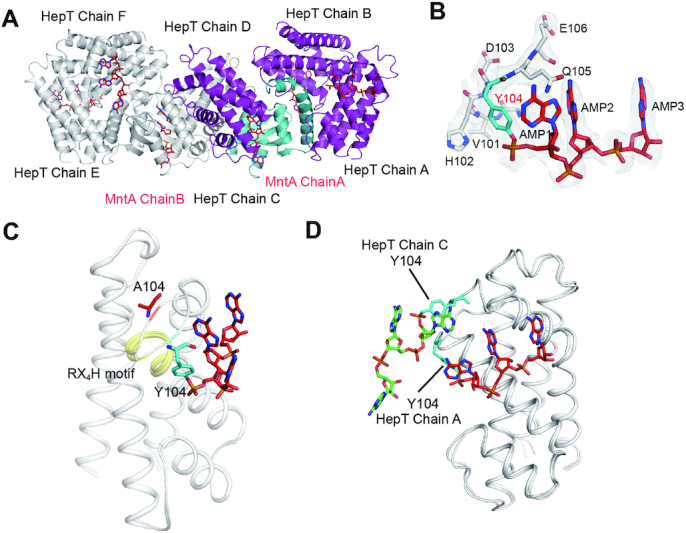
Structural basis of MntA mediated polyadenylylation of HepT. (**A**) Crystal structure of the hetero-octamer HepT/MntA complex. MntA and HepT are shown as cartoons, and the colored structure shows one ASU containing three HepTs (purple) and one MntA (cyan). (**B**) Electron density map of the modified Y104 and the three AMP moieties. The 2*F*o – *F*_c_ omit map was contoured at the 1.0σ level. (**C**) Structural superposition of polyadenylylation and un-modified HepT cells. The conserved RX_4_H motif is highlighted in yellow. (**D**) The superposition of HepT chain A and HepT chain C.

To further verify the chemical modification of HepT directly by MntA, protein samples of the *S. oneidensis* wild-type (WT) HepT/MntA complex and its derivative complex were prepared by co-expression of the corresponding proteins. We again observed a shift in HepT in the WT HepT/MntA complex when compared with the HepT^Y104A^/MntA complex in the SDS-PAGE gel (Figure 1BC), suggesting that HepT was modified automatically when co-expressed in *E. coli*. We determined the crystal structures of HepT/MntA and HepT^Y104A^/MntA at 3.08 and 2.6 Å, respectively (Table 1). There were four molecules composed of one MntA polypeptide and three HepT polypeptides in one asymmetric unit (ASU), and the complex formed a hetero-octamer based on symmetry analysis (Figure 3A); this result is consistent with our previous report (17). Similarly, a (HepT)_6_-(MntA)_2_ hetero-octamer was found in the ASU of HepT^Y104A^/MntA (Supplementary Figure S6). During structure determination, three regions of the unambiguous electron map connected to the hydroxyl group of Y104 in HepT were exclusively observed in the structure of WT HepT/MntA. In view of the above mentioned results, antitoxin MntA polyadenylylates toxin HepT, and in the final structure, a total of three AMP moieties linked by 3′-5′ phosphodiester bonds were accommodated in every region of the additional electron density map (Figure 3B). In contrast, this additional electron density map was no longer present in the HepT^Y104A^/MntA complex (Supplementary Figure S6). Y104 of HepT was polyadenylylated, and a phosphodiester bond was formed by the α-phosphate of the first AMP (AMP1) and the hydroxyl group of Y104 (Figure 3B). The MntA molecule in HepT/MntA was almost identical to its counterpart in HepT^Y104A^/MntA, with a maximal root mean square deviation (RMSD) of 0.26 Å over 98 Cα and 0.29396 Cα, respectively. Comparison of the polyadenylylated HepT and unmodified HepT^Y104A^ revealed that they were nearly identical with an RMSD of 0.396 Å over the 953 Cα main chain, except for the loop containing Y104, which was rotated clockwise nearly 180° (Figure 3C). Interestingly, detailed analysis of the AMP triplet moieties of the three HepT molecules in the ASU revealed that the orientation on Chains A or B was different from the one on Chain C. For clarity, the polyadenylylated structures of chain A of HepT and chain C of HepT were extracted (Figure 3D). The extraction showed that the core of their structures aligned well, except for where the loops of the AMP chains were located. The AMP moiety in Chain A was twisted toward the cleft formed by α4 and α5 of HepT. In contrast, the AMP moieties in chain C of HepT were oriented to MntA (Figure 3D). Collectively, our structural analysis confirm that Y104 of HepT is polyadenylylated.

### MntA transfers three AMPs to HepT consecutively using ATP as the substrate

**Figure 4.**
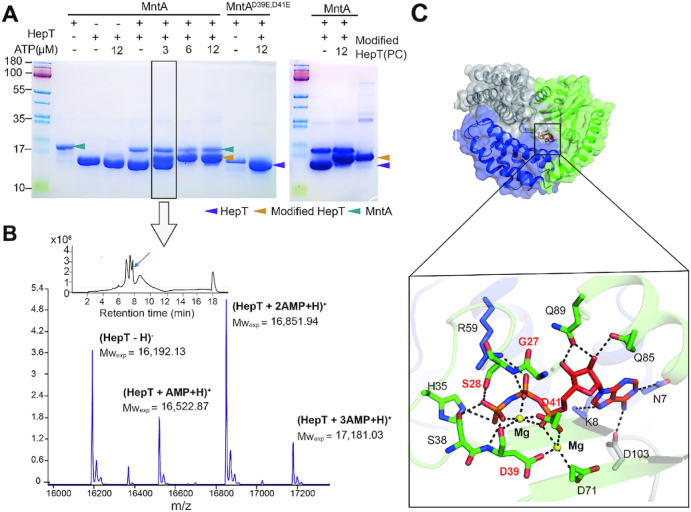
MntA mediates the transfer of three AMPs to HepT consecutively. (**A**) MntA modified HepT in the presence of ATP.As a negative control, MntA^D39E, D41E^ was also tested under the same conditions (left panel). The fully reacted HepT was compared with the purified modified HepT which serves as a positive control (PC) in the right panel. (**B**) The experimentally identified MWs of HepT incubated with MntA and 3 μM ATP. The reaction in panel A (black box) were stopped by adding EDTA. Then the protein mix were analyzed by HPLC/Q-TOF-MS. The MS spectra of HepT peaks (marked with blue arrow) are shown at a retention time of 7.738–7.854 min. (**C**) Structure of HepT^Y104A^/MntA in complex with AMP-PNP. The AMP–PNP substrate analog, shown as sticks in red, is bound in the cleft formed between the MntA and the HepT^Y104A^. Close-up view of the interaction between AMP-PNP and HepT^Y104A^/MntA; the residues involved in binding HepT^Y104A^/MntA are shown in sticks. Hydrogen bonds are shown as black dashed lines.

To elucidate the molecular mechanism of polyadenylylation mediated by MntA, we performed an *in vitro* enzymatic assay using purified MntA and HepT (unmodified). The result showed that MntA mediated the modification of HepT using ATP as a substrate (left panel, Figure 4A), and the size of the HepT obtained in this reaction fits well with size found for modified HepT *in vivo* (right panel, Figure 4A). As expected, when the key active motif GSX_10_DXD of MntA is mutated, it can no longer modify HepT (left panel, Figure 4A). Additionally, in the presence of biotin-17-ATP as the substrate, when purified MntA was incubated with purified HepT or when purified MntA was incubated with *E. coli* cell lysate containing HepT, biotin-labeled HepT was detected using anti-biotin antibodies and western blot analysis (Supplementary Figure S7A). Moreover, previous work indicated the Mg^2+^ ions are needed for adenylyltransferase activity, so we tested this requirement and found that the *in vitro* adenylylation assay requires Mg^2+^ ions (Supplementary Figure S7B). Therefore, MntA modifies HepT via ATP through its GSX_10_DXD motif and requires Mg^2+^ as is typical for adenylyltransferase activity.

Since few adenylyltransferases can mediate the transfer of more than one AMP to the target, we thus investigated whether the three AMPs were added to HepT consecutively by analyzing the intermediate products of HepT modification by MntA. To obtain intermediates, the reaction was stopped by adding EDTA, and the products were analyzed by HPLC/Q-TOF-MS. The MS analysis showed that four types of HepT proteins were identified, including unmodified HepT and HepT proteins with one, two or three AMPs (Figure 4B). These results clearly show that MntA mediates the transfer of the three AMPs to HepT in a consecutive fashion using ATP as the substrate.

In addition, to understand the adenylylation reaction at the atomic scale, we determined the crystal structure of HepT^Y104A^/MntA in complex again but this time with the non-hydrolysable ATP analogue adenylyl imidodiphosphate (AMP–PNP) at 2.77 Å. The structure reveals that AMP–PNP is in the cavity formed between MntA and HepT^Y104A^, with the phosphate group of AMP-PNP inserted into the GSX_10_DXD motif (Figure 4C). Multiple interactions facilitate AMP–PNP binding. Specifically, the γ -phosphate of AMP–PNP bonds with S38, D39 and S28 of MntA, and the β-phosphate of AMP-PNP forms hydrogen bonds with R59 of HepT and G27, S28 of MntA. In addition, the adenosine engages in hydrogen-bonding with N7, D103 of HepT and Q85, Q87 of MntA. There are two Mg^2+^ ions in every catalytic center of MntA, with one Mg^2+^ ion is coordinated by the oxygen atoms of the α, β and γ phosphate group and D39, D41 of MntA and the other Mg^2+^ ion is coordinated by oxygen atom of the α phosphate group and D41, D39 and D71 of MntA. Therefore, the HepT^Y104A^/MntA structure with AMP-PNP shows how MntA utilizes ATP and Mg^2+^ for the adenylylation reaction with HepT.

To further confirm the role of the conserved MntA GSX_10_DXD motif in mediating adenylylation, the structure of the HepT/MntA^D39E, D41E^ hetero-octamer was determined at 2.35 Å (Supplementary Figure S6). The structural analysis confirmed that HepT was not adenylylated even though Y104 was available for receiving AMP. Taken together, we demonstrated that MntA transfers three AMPs to the Y104 residue of HepT by employing a highly conserved GSX_10_DXD motif.

### Neutralization of HepT by MntA relies on polyadenylylation

**Figure 5.**
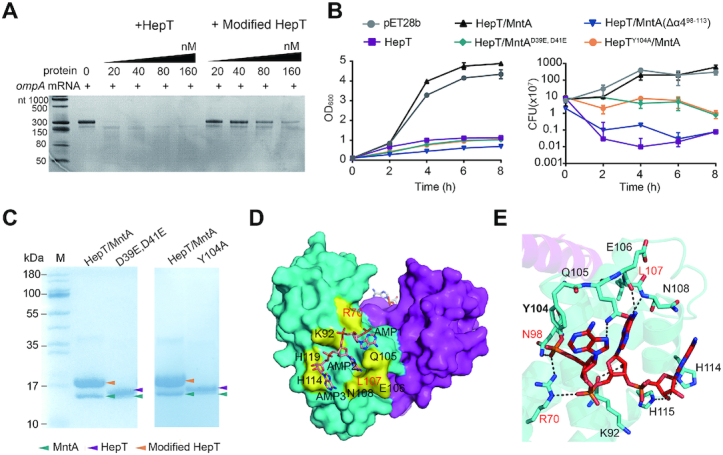
Polyadenylylation is critical for MntA to neutralize HepT. (**A**) Unmodified HepT cleaved *ompA* mRNA but polyadenylylated HepT has a reduced RNase activity. (**B**) Growth and CFU of *E. coli* BL21 carrying pET28b-based plasmids were determined after the addition of 0.5 mM IPTG at an OD_600_ of 0.1. Since HepT is a RNase toxin, production of HepT under physiological conditions should stop growth instead of killing cells, thus the drop in CFU seen from HepT overproduction was caused by using the strong RBS in pET28b-*hepT*. For these experiments, the native HepT/MntA and HepT/MntA variants should have similar HepT production as they use the native RBS for HepT in the pET28b-*mntA*-*hepT*constructs. Three independent cultures of each strain were tested, and error bars indicate the standard error of the mean (*n* = 3). (**C**) HepT/MntA complexes and its variants HepT/MntA^D39E, D41E^, HepT^Y104A^/MntA were expressed from pET28b-based plasmids in *E. coli* BL21 and purified. (**D**) Structural analysis of polyadenylylated dimeric HepT. HepT is shown in surface mode, and the AMP moieties are shown as sticks. The residues that aided in stabilizing the AMP moieties are highlighted in yellow. (**E**) The interaction between HepT and the AMP moiety. The residues involved in hydrogen bonding are shown as sticks.

Based on the conserved signature RX_4_HXY motif in the toxin component and the signature GSX_10_DXD motif in the antitoxin component, we reasoned that polyadenylylation likely plays an important role in the neutralization mechanism of this TA pair. We previously demonstrated that HepT is an RNase that cleaves mRNA substrates *in vitro* (17). Compared to the type II TA pair ParE_SO_/CopA_SO_, MntA and HepT have a weaker interaction *in vivo* (Supplementary Figure S8). Based on this weaker interaction, the modified HepT protein was successfully purified from the lysate of cells co-expressing the TA complex (Supplementary Figure S1). The *in vitro* RNase activity of the modified HepT and unmodified HepT proteins was measured with *ompA* mRNA as the substrate. The results clearly revealed that polyadenylylated HepT did not cleave mRNA as effectively as unmodified HepT (Figure 5A). Since the toxicity of HepT can be measured by cell growth (OD_600_) and cell viability (CFU/ml)(17), we performed a toxicity test using HepT/MntA native to *S. oneidensi*s and HepT and MntA variants. As shown previously, overexpressing MntA did not induce toxicity (17). Although substitution of Y104 in the RX_4_HXY motif did not affect the toxicity of HepT (16), this substitution clearly reduced the ability of MntA to neutralize HepT (Figure 5B). The substitutions of DXD with EXE in the GSX_10_DXD motif also reduced the ability of MntA to neutralize HepT (Figure 5B). Thus, both *in vitro* and *in vivo* assays demonstrate that polyadenylylation is important for reducing HepT toxicity.

Since MntA can bind to HepT and polyadenylylate HepT, we wanted to check the importance of protein-protein interactions and/or adenylylation in terms of neutralizing HepT toxicity. The substitutions of MntA (G27/S28 or D39/D41) showed a reduced ability to neutralize HepT toxin (Figure 5B and Supplementary Figure S9). However, our structural analysis clearly showed that the MntA^D39E, D41E^ variant binds to HepT to form a hetero-octamer in a way that is like native MntA (Supplementary Figure S6). Additionally, the MntA variants MntA^D39E, D41E^ are still pulled down due to their strong protein-protein interaction with HepT during SDS-PAGE fractionation of the TA complex (Figure 5C). These results collectively suggest that the binding to HepT is not sufficient for MntA to neutralize the toxicity of HepT. In our previous study, we found that MntA binds to HepT mainly via the insertion of the α4 helix into the HepT RX_4_H motif, and the deletion of the α4 helix of MntA (MntA Δα4^98–113^) reduced the ability to neutralize the toxicity of HepT (Figure 5B) (17). We then tested whether the deletion of the α4 helix of MntA affects adenylylation using HepT/MntA Δα4^98–113^ (Supplementary Figure S10). Noticeably, unmodified HepT was observed in the presence of MntA Δα4^98–113^ but not in the presence of native MntA, suggesting that the α4 helix of MntA is needed for the full adenylyltransferase activity. To probe the molecular mechanism of how polyadenylylation affects the toxicity of HepT, we analyzed the polyadenylylated and unmodified dimeric forms of HepT. The analysis of the interaction of HepT and the AMP moieties revealed that AMP fits near the cavities along the cleft access to the RX_4_H RNase activity site (Figure 5D). Furthermore, the highly conserved R70 that was located in the positively charged region of HepT forms hydrogen bonds with the first AMP moiety (Figure 5E). This result is in agreement with our previous study that substitution of R70 with H70 or substitution of L107 with A107 in HepT abolished HepT toxicity (16). Collectively, these results reveal that the neutralization of MntA mainly depends on polyadenylylation.

### HepT/MntA TA systems are ubiquitously distributed in prokaryotes

**Figure 6.**
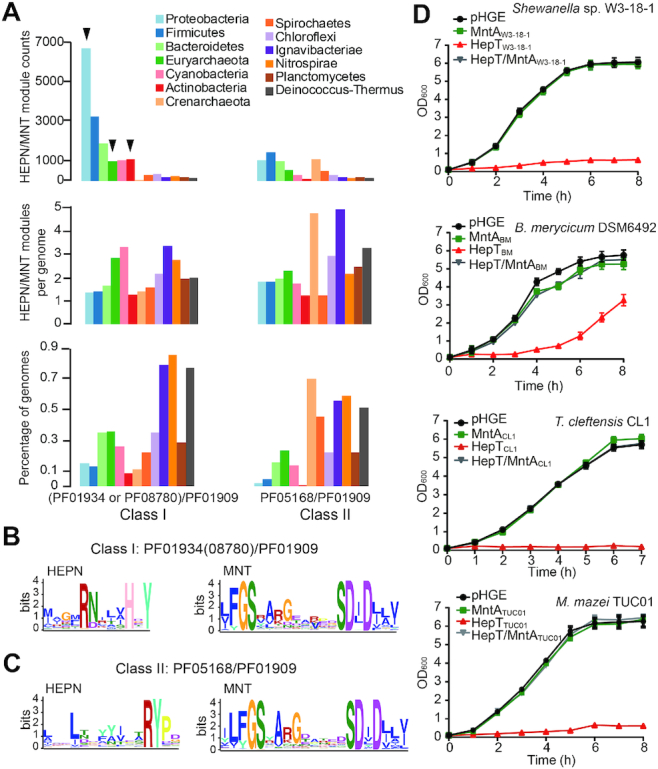
Prevalence of HEPN/MNT in bacteria and archaea. (**A**) Distribution of the Class I (PF01934 or PF08780/PF01909) and Class II (PF05168/PF01909) HEPN/MNT modules in representative phyla. The total number (top), the average number per genome (middle); and the percentage of genomes identified (bottom) are shown for Class I and Class II. The HEPN/MNT pairs chosen from the phyla for characterization in panel D are marked with black inverted triangles. Sequence profile of the conserved motif in the Class I (**B**) and Class II (**C**) HEPN/MNT modules. A total of 19, 469 sequences of Class I HEPN/MNT modules and 9, 583 sequences of Class II HEPN/MNT modules were used for motif scanning. (**D**) Growth of *E. coli* K12 carrying pHGE-based plasmids was determined after the addition of 0.5 mM IPTG at an OD_600_ of 0.1. The HEPN/MNT pairs chosen including the deep-sea bacterium *S. putrefaciens* W3-18-1, the rumen bacterium *Bifidobacterium merycicum* DSM6492, the archaeon *Thermococcus cleftensis* CL1, and the methanogenic archaeon *Methanosarcina mazei* TUC01. Three independent cultures of each strain were tested, and error bars indicate the standard error of the mean (*n* = 3).

Since HEPN domain-containing and MNT domain-containing proteins are widespread in bacteria and archaea (13), we searched for HEPNs encoded next to MNTs using the conserved GSX_10_DXD and RX_4_HXY motifs by FIMO (29). The majority (99%) of neighboring HEPN/MNT domains belonged to the HEPN homologous domains (PF01934: DUF86 or PF08780: NTase_sub_bind) and the MNT domain (PF01909: NTP_transf_2) according to the Pfam database. Because another important HEPN homologous domain (PF05168: HEPN) was also found to flank the MNT-domain PF01909 family (14), these two classes of HEPN/MNT pairs were recovered from the IMG/M database with functional profile tools (Supplementary Tables S3 and S4). We found that the Class II HEPN/MNT module (PF05168/PF01909) was more prevalent than the Class I HEPN/MNT module (PF01934 or PF08780)/PF01909 in archaea, similar to the report by Makarova and Koonin (14). Moreover, we found that Class I pairs were much more prevalent than Class II pairs in bacteria, with Class I pairs found in nearly 15% of Proteobacteria genomes (Figure 6A and Supplementary Figure S11). Interestingly, the antitoxin components of the Class I and II modules both contain the conserved motif GSX_10_DXD, suggesting that nucleotidyltransferase activity is conserved in these antitoxins. In contrast, the conserved motif RX_4_HXY in HepT was only found in the toxin component of the Class I module (Figure 6BC). Furthermore, a conserved tyrosine, which can be adenylylated, was also found in the toxin of the Class II TA module, although no active RNase motif RX_4_H was found (Figure 6C). Collectively, these results suggest that these HEPN/MNT modules represent two different TA families that may differ in the catalytic function of the toxin component. To further test whether the HEPN domain-containing proteins are toxins in the Class I TA modules, four putative TA pairs from Class I were selected: from the deep-sea bacterium *Shewanella putrefaciens* W3-18-1; from the archaeon *T. cleftensis* CL1, which was isolated from a deep-sea hydrothermal sulfide chimney; from the methanogenic archaeon *Methanosarcina mazei* TUC01, a strain isolated from an Amazon flooded area; and from *Bifidobacterium merycicum*, which was isolated from the rumen of cattle (Supplementary Table S6 and Figure S3). Consistently, in these HEPN/MNT modules, the HEPNs serve as the toxin, and the upstream MNT domain-containing proteins completely neutralize the toxicity of the cognate toxins (Figure 6D and Supplementary Figure S12). Collectively, these results demonstrate that HEPNs with the RX_4_HXY motif and neighboring MNTs with the GSX_10_DXD motif represent a new type of TA system that is prevalent in both bacteria and archaea.

### HepT is adenylylated by MntA in the archaeon *T. cleftensis*

**Figure 7.**
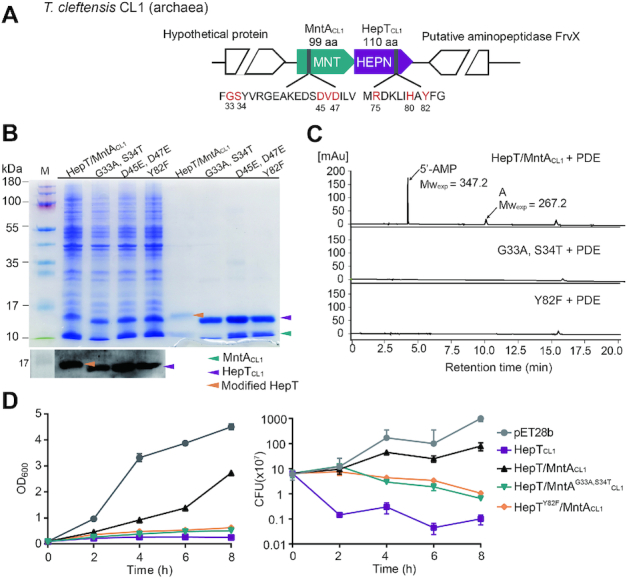
HepT_CL1_ is adenylylated by MntA_CL1_ in *T. cleftensis* CL1. (**A**) Location of the HepT_CL1_ (CL1_0071)/MntA_CL1_ (CL1_0070) pair and neighboring genes in the *T. cleftensis* genome. (**B**) His-tagged HepT_CL1_ and untagged MntA_CL1_ were induced from the pET28b-based plasmid in *E. coli* BL21 and co-purified (lanes 2 and 6). HepT_CL1_/MntA_CL1_ mutants were also induced and co-purified using the pET28b-based plasmid in *E. coli* BL21. Western blot (lower panel) was performed using monoclonal anti-His tag antibodies for the same set of samples. (**C**) HPLC-MS analysis of the molecules digested from the indicated TA complex by PDE nuclease. (**D**) Growth and CFU of *E. coli* BL21 carrying pET28b-based plasmids were determined after the addition of 0.5 mM IPTG at OD_600_ of 0.1. Three independent cultures of each strain were tested, and error bars indicate the standard error of the mean (*n* = 3).

To further investigate whether the antitoxin component of the Class I HEPN/MNT TA module can adenylylate the toxin in archaea, the HepT/MntA pair in *T. cleftensis* CL1 was chosen (Figure 7A). *T. cleftensis* CL1 is a hyperthermophilic archaeon isolated from a *Paralvinella* sp. polychaete worm living on the deep-sea hydrothermal sulfide chimney of the Juan de Fuca Ridge (33). Overexpressing HepT_CL1_ in *E. coli* led to severe growth inhibition, and co-expressing neighboring MntA_CL1_ completely neutralized the toxicity of HepT_CL1_ (Figure 6D and Supplementary Figure S12). Similarly, site-directed mutagenesis was utilized to generate two MntA_CL1_ variants with the conserved GSX_10_DXD motif substituted with ATX_10_DXD or GSX_10_EXE. One HepT_CL1_ variant with a substitution at Y82 in the RX_4_HXY signature motif was also constructed. As expected, substitution of Y82 with F82 in the RX_4_HY motif of HepT _CL1_ or substitution of G33S34 with A33T34 in the GSX_10_DXD motif of MntA_CL1_ eliminated the ability of MntA_CL1_ to modify HepT_CL1_ (Figure 7B). Additionally, HPLC/MS analysis showed that the AMP peak only existed in the cleaved products of HepT/MntA_CL1_ (Figure 7BC and Supplementary Figure S13). Substitution of the GSX_10_DXD motif to ATX_10_DXD in MntA_CL1_ or the substitution of Y to F in the RX_4_HXY motif of HepT_CL1_ both reduced the ability of the antitoxin to rescue the growth inhibition of HepT (Figure 7D). Taken together, these results suggest that HEPN proteins with the RX_4_HXY motif and the neighboring MNT-containing protein with the GSX_10_DXD motif form a TA pair in archaea, and the adenylylation of the toxin by the GSX_10_DXD signature motif of the antitoxin might represent a common mechanism for this TA pair.

## DISCUSSION

**Figure 8.**
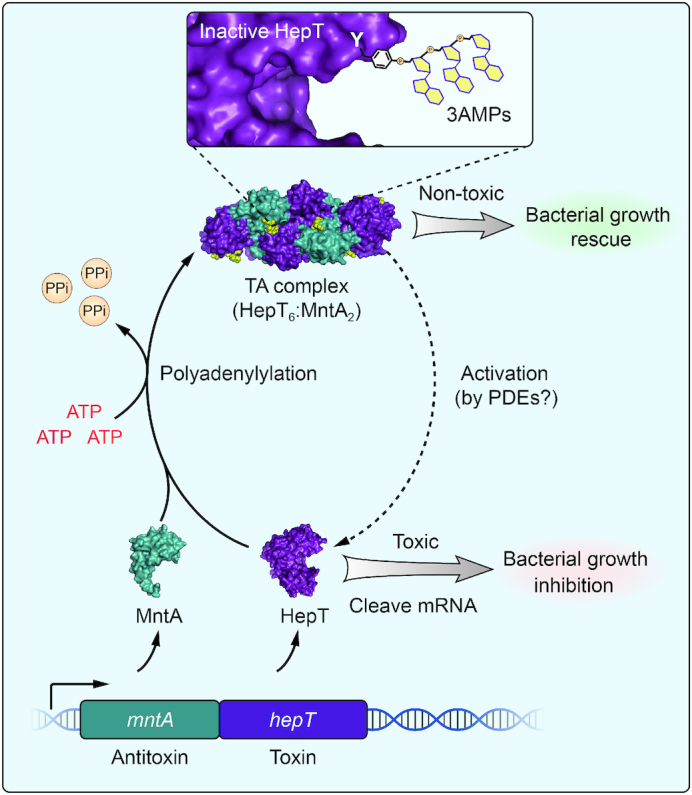
A proposed model of the neutralization mechanism of the HepT/MntA TA system. Unmodified HepT can cleave cellular mRNAs, leading to bacterial growth arrest. MntA can inactive the HepT toxin by transferring three AMPs to Y104 in the RX_4_HXY motif of HepT, thus inhibiting the catalytic activity of HepT. The gray dashed arrow indicates the reversal of anti-toxicity, which may require unknown PDEs.

The HEPN/MNT module is predicted to be one of the most abundant TA pairs and is prevalent in various bacterial and archaeal strains (34). Here, we provide evidence that the HEPN/MNT module is a new type of TA system (HepT/MntA) in both bacteria and archaea. Currently, six different types of TA systems have been reported and recognized. Recently, three examples of antitoxins inactivating toxins as enzymes have been found. In the newly proposed type VII Hha/TomB system in *E. coli*, the antitoxin TomB oxidizes the cysteine residues of toxin Hha to neutralize Hha toxicity (10). In the TglT/TakA system in M*ycobacterium tuberculosis*, the antitoxin TakA functions as a serine protein kinase that inactivates toxin TglT by phosphorylating it at residue Ser78 (12). We found here that MntA antitoxin acts as an adenylyltransferase and catalyzes the transfer of three AMP moieties onto toxin HepT to block its toxicity, representing a new type of interaction between the toxin and the antitoxin (Figure 8). Thus, three enzymatic antitoxins TomB, TakA and MntA chemically modify the toxin to neutralize its toxicity. Therefore, we propose to classify HepT/MntA TA pair as a type VII TA system in which the neutralization mechanism relies on the enzymatic activity of the antitoxins in a similar way as Hha/TomB (35).

A typical feature of type II TA systems is the tight affinity between the toxin and the antitoxin component, which key to the neutralization mechanism. In the Hha/TomB TA pair, there was only a transient affinity between the toxin and antitoxin (10). Although a hetero-octamer of HepT/MntA was found in *S. oneidensis*, we found that the affinity between HepT and MntA was lower than that of a typical type II TA system. Here, we also explored the importance of the enzymatic activity of MntA and the involvement of protein-protein interactions in terms of the neutralization mechanism. Both the *in vitro* and *in vivo* assays demonstrated that polyadenylylation is more important for reducing HepT toxicity than the affinity between HepT and MntA. Structural analysis of HepT^Y104A^/MntA in complex with AMP–PNP also revealed that the AMP–PNP substrate is not in the proximity of Tyr104 in HepT, and it is possible that the HepT/MntA hetero-octamer is not the only state of this TA pair in the cell. We also noticed that the amount of MntA protein was equally expressed in general as the toxin; thus, free MntA is likely present in the cell and might be involved in HepT modification. Furthermore, our bioinformatics analysis of class I HEPN/MNT TA modules in different bacteria and archaea revealed a conserved motif with adenylyltransferase activity and a conserved Y critical for receiving AMPs, suggesting that the chemical modification of the toxin by its antitoxin is a common neutralization mechanism in these putative TA pairs.

Previously, toxins with nucleotidyltransferase activity have been reported for the FicT/FicA (36) and AbiEii/AbiEi (37) TA systems. We report here that an antitoxin can also function as a nucleotidyltransferase. The conserved motif in MNT antitoxins is GSX_10_DXD which is in agreement of the conserved motif (GX_11_DXD) in the previously reported AMPylators with the adenylyltransferase domain (38). The first AMPylating protein with an adenylyltransferase domain reported was glutamine synthetase adenylyltransferase (GS-ATase) in *E. coli* that mediates the transfer of an AMP to the glutamine synthetase in response to changes in nitrogen (39–42). Later, the *Legionella pneumophila* effector protein DrrA (or SidM) was reported to AMPylate the membrane traffic regulator GTPase Rab1 (43). Unlike GS-ATase and DrrA, which contain more than one domain (>300 aa), MNTs are <150 aa. To the best of our knowledge, MntA is the first adenylyltransferase that can mediate the transfer of three AMPs to the target consecutively. Mutagenesis experiments revealed that the GSX_10_DXD motif of MntA is the key for adding three AMP moieties to Y104 of HepT. The structural analysis showed that the Y104 residue near the active RX_4_H of HepT receives the three AMP moieties, thus this residue may hinder the access of HepT to its RNA substrate. This is consistent with previously reported AMPylators which can modulate the activity of modified targets by interfering with substrate binding (36,44).

The understanding of the regulatory role of adenylylation in prokaryotes is in its infancy, and many questions about the biological processes it participates in remain to be answered. Furthermore, two different classes of HEPN/MNT pairs have been found, and their abundance in bacteria and archaea is different. Class II HEPN/MNT modules were first predicted by Koonin's group and have a wide distribution in the hyperthermophilic bacteria and archaea; yet, no biochemical studies on this module have been conducted as far as we know (14). Our work here shows the Class I HepT/MntA pair in archaeon *T. cleftensis* CL1 works through adenylylation. However, the HEPNs in Class II do not have the RNase RX_4_HXY domain, but the neighboring MNTs still have the conserved motif GSX_10_DXD. Thus, further study is needed to validate whether they constitute a TA system and to investigate the adenylylation mechanism in archaea. In addition, understanding the conditions that trigger adenylylation and/or de-adenylylation of the toxin is a critical question that needs to be addressed in future studies of HEPN/MNT modules to understand the physiological role of these TA systems. Potential PDEs that can remove AMPs from the modified HepT to reactivate the toxin have yet to be identified. Nevertheless, the prevalence of these HEPN/MNT modules in bacteria and archaea isolated from diverse environments provides an opportunity to learn more about chemical modification and biological processes in different domains of life.

## DATA AVAILABILITY

Atomic coordinates and structure factors have been deposited in the Protein Data Bank (PDB) with the following codes: HepT/MntA (6M6V), HepT/MntA^D39E, D41E^ (6W6U), HepT^Y104A^/MntA (6M6W) and HepT^Y104A^/MntA-AMP-PNP (7BXO).

## Supplementary Material

gkaa855_Supplemental_Files
