# Supplementary material for: Novel polyadenylylation-dependent neutralization mechanism of the HEPN/MNT toxin/antitoxin system
Source: Nucleic Acids Res. 2020 Oct 12;48(19):11054–67. doi: 10.1093/nar/gkaa855 (PMC7641770; doi:10.1093/nar/gkaa855)
Supplement: gkaa855_Supplemental_Files [file gkaa855_supplemental_files.zip › SI File 09-18-2020v1.docx]

**Supplementary File**

**Novel** **Polyadenylylation-Dependent Neutralization Mechanism of the HEPN/MNT Toxin/Antitoxin System**

**List of Supplementary Materials in this PDF file:**

- Tables S1, S2, S5, S6
- Figures S1-S13
- References

**Other Supplementary Materials uploaded separately:**

- Table S3-S4 is uploaded as one separate excel file
- Structural files (in zip file): the PDB validation report (.pdf); the molecular coordinates (.pdb); X-ray data (.mtz)

**For the four structures:**

1. HepT/MntA

2. HepT^Y104A^/MntA

3. HepT/MntA^D39E, D41E^

4. HepT^Y104A^/MntA bound with AMP-PNP

**Table S1.** Bacterial strains and plasmids used in this study.

| **Bacterial strains/Plasmids** | **Description^a^** | **Source** |
| --- | --- | --- |
| **strains** |  |  |
| BL21(DE3) | F*^-^ompT hsdS_B_(r_B_^-^m_B_^-^) gal dcm λ*(DE3) Ω P_tacUV5_::T7 polymerase | Novagen |
| K-12 BW25113 | *lacI*^q^ *rrnB*_T14_ Δ*lacZ*_WJ16_ *hsdR*514 Δ*araBAD*_AH33_ Δ*rhaBAD*_LD78_ | ((1)) |
| WM3064 | *thrB*1004 *pro thi rpsL hsdS lacZ*ΔM15 RP4-1360) Δ(*araBAD*)567 Δ*dapA*1341::[*erm* *pir(*wt)] | W. Metcalf, UIUC |
| *S.oneidensis* | *S. oneidensis*, wild type | Lab stock |
| **Plasmids** |  |  |
| **pHGE** | pHGE-Ptac, Km^R^, IPTG inducible expression plasmid | (2) |
| pHGE-*hepT-flag* | Km^R^; expression plasmid for *hepT-flag* in MR-1 | this study |
| pHGE-*mntA-hepT-flag* | Km^R^; expression plasmid for *mntA-hepT-flag* of MR-1 | this study |
| pHGE-*mntA*^D39E,D41E^*-hepT-flag (D39E,D41E)-3166-flag*  *-flag* | Km^R^; expression plasmid for *mntA*^D39E,D41E^*-hepT-flag* in MR-1 | this study |
| pHGE-*mntA-hepT*^Y104A^*-flag* | Km^R^; expression plasmid for *mntA-hepT*^Y104A^*-flag* in MR-1 | this study |
| pHGE-*mntA*_CL1_ | Km^R^; expression plasmid for *mntA*_CL1_ in *E. coli* | this study |
| pHGE-*hepT*_CL1_ | Km^R^; expression plasmid for *hepT*_CL1_ in *E. coli* | this study |
| pHGE-*mntA*-*hepT*_CL1_ | Km^R^; expression plasmid for *mntA*-*hepT*_CL1_ in *E. coli* | this study |
| pHGE-*mntA*_W3-18-1_ | Km^R^; expression plasmid for *mntA*_W3-18-1_ in *E. coli* | this study |
| pHGE-*hepT* _W3-18-1_ | Km^R^; expression plasmid for *hepT* _W3-18-1_ in *E. coli* | this study |
| pHGE-*mntA*-*hepT*_W3-18-1_ | Km^R^; expression plasmid for *mntA*-*hepT*_W3-18-1_ in *E. coli* | this study |
| pHGE-*mntA*_TUC01_ | Km^R^; expression plasmid for *mntA*_TUC01_ in *E. coli* | this study |
| pHGE-*hepT*_TUC01_ | Km^R^; expression plasmid for *hepT*_TUC01_ in *E. coli* | this study |
| pHGE-*mntA*-*hepT*_TUC01_ | Km^R^; expression plasmid for *mntA*-*hepT*_TUC01_ in *E. coli* | this study |
| pHGE-*mntA*_DSM6492_ | Km^R^; expression plasmid for *mntA*_DSM6492_ in *E. coli* | this study |
| pHGE-*hepT* _DSM6492_ | Km^R^; expression plasmid for *hepT* _DSM6492_ in *E. coli* | this study |
| pHGE-*mntA*-*hepT* _DSM6492_ | Km^R^; expression plasmid for *mntA*-*hepT* _DSM6492_ in *E. coli* | this study |
| **pET28b** | Km^R^, *lacI*^q^, IPTG inducible expression plasmid | Novagen |
| pET28b-*hepT-*His | Km^R^, *lacI*^q^, P*_T7-lac_*::*hepT* with *hepT* C-terminus His-tagged | this study |
| pET28b-*mntA-hepT*-His | Km^R^, *lacI*^q^, P*_T7-lac_*::*mntA-hepT* with *hepT* C-terminus His-tagged | (3) |
| pET28b-*mntA^G27A,S28A^-hepT*-His | Km^R^, *lacI*^q^, P*_T7-lac_*:: *mntA^G27A,S28A^-hepT* with *hepT* C-terminus His-tagged | this study |
| pET28b-*mntA^D39E,D41E^-hepT*-His | Km^R^, *lacI*^q^, P*_T7-lac_*:: *mntA^D39E,D41E^-hepT* with *hepT* C-terminus His-tagged | this study |
| pET28b-*mntA-hepT*^Y104A^-His | Km^R^, *lacI*^q^, P*_T7-lac_*:: *mntA-hepT^Y104A^* with *hepT* C-terminus His-tagged | this study |
| pET21-*mntA*-His | Amp^R^, *lacI*^q^, P*_T7-lac_*:: *mntA* with N-terminus His-tagged | this study |
| pET28b-*mntA-hepT*_CL1_-His | Km^R^, *lacI*^q^, P*_T7-lac_*:: *mntA-**hepT*_CL1_ with *hepT*_CL1_ C-terminus His-tagged | this study |
| pET28b-*mntA^G33A,S34A^-hepT*_CL1_-His | Km^R^, *lacI*^q^, P*_T7-lac_*:: *mntA^G33A,S34A^-hepT*_CL1_ with *hepT*_CL1_ C-terminus His-tagged | this study |
| pET28b-*mntA^D45E,D47E^-hepT*_CL1_-His | Km^R^, *lacI*^q^,P*_T7-lac_*:: *mntA^D45E,D47E^-hepT*_CL1_ with *hepT*_CL1_ C-terminus His-tagged | this study |
| pET28b-*mntA-hepT*^Y82F^-His | Km^R^, *lacI*^q^, P*_T7-lac_*:: *mntA-hepT*^Y82F^ with *hepT*^Y82F^ C-terminus His-tagged | this study |
|  |  |  |

^a^ Cm^R^, Km^R^, Gm^R^ and Amp^R^ indicate chloramphenicol, kanamycin, gentamycin, and ampicillin resistance.

**Table S2.** Oligonucleotides used for plasmid construction and DNA sequencing. Restriction enzyme sites and vector sequence for one-step are underlined. f indicates forward primer and r indicates reverse primer.

| **Purpose/Name** | **Sequence (5'-3')** |
| --- | --- |
| **Primers for characterizing TA systems** | |
| pHGE-*mntA*_CL1_*-*f | CAATTTCACACAGGAGAGAATTCATGGCAATGCTGACCCTTGAAG |
| pHGE-*mntA*_CL1_*-*r | CATCCGCCAAAACAGCCAAGCTTGGATCCTCATACATAGACTACCTCCCTG |
| pHGE-*hepT*_CL1_-f | CAATTTCACACAGGAGAGAATTCATGAAACGCTCTCATAAGGA |
| pHGE-*hepT*_CL1_*-*r | CATCCGCCAAAACAGCCAAGCTTGGATCCCTACTTCCTAAGCTTCTCAAAT |
| pHGE-*mntA*_TUC01_-f | CAATTTCACACAGGAGAGAATTCATGGATAAGAGTTCATTTCTGGCA |
| pHGE-*mntA*_TUC01_-r | CCGCCAAAACAGCCAAGCTTGGATCCTTAGACCCTCGCCAGGAATTCC |
| pHGE-*hepT*_TUC01_-f | CAATTTCACACAGGAGAGAATTCATGAGAATACTTGCCCATTTA |
| pHGE-*hepT*_TUC01_-r | CCGCCAAAACAGCCAAGCTTGGATCCTCATTCAGTAGATAAATCCTGA |
| pHGE-*mntA*_W3-18-1_-f | CCGGAATTCATGACGATCGAACAGAGCCAGC |
| pHGE-*mntA*_W3-18-1_-r | CGCGGATCCTTATAGGCTCTGAGCAAAGTA |
| pHGE-*hepT*_W3-18-1_-f | CCGGAATTCATGTCTGATAATGCCTATATCACATC |
| pHGE-*hepT*_W3-18-1_-r | CGCGGATCCTTAGCTATTTTTCAAAGATAACCA |
| PHGE-*mntA*_DSM6492_*-f* | CAATTTCACACAGGAGAGAATTCATGAAGCGTGGATTGAGCCAACA |
| PHGE-*mntA*_DSM6492_*-r* | CATCCGCCAAAACAGCCAAGCTTGGATCCTCACGCCACCTGCACCATGTCA |
| PHGE-*hepT* _DSM6492_*-f* | CAATTTCACACAGGAGAGAATTCATGGCGAGGATTCATGCGGATCA |
| PHGE-*hepT* _DSM6492_*-r* | CATCCGCCAAAACAGCCAAGCTTGGATCCTCAATCTTCAATAGCTTCGACGT |
| **Primers for site directed mutagenesis using fusion PCR** | |
| *mntA^G27A, S28A^*-f | TATTCGCAGGGAACACAACACCG |
| *mntA^G27A, S28A^*-r | CCTGCGAATATGCTGCAAACAGGT |
| *mntA^D39E, D41E^*-f | ATCGCGGTACTCGCCGCTGACACT |
| *mntA^D39E, D41E^*-r | GTACCGCGATCTCGATCTCGCTGT |
| *HepT^Y104A^-r* | CAAGAACTCAACCTCGATA |
| *HepT^Y104A^-r* | GAGTTCTTGGGCGTCATGTACT |
| *mntA*_CL1_*^G33A, S34T^*-f | TACGTCCGGGGAGAGGCAAAGG |
| *mntA*_CL1_*^G33A, S34T^*-r | CCGGACGTAAGTGAGAAACACCC |
| *mntA*_CL1_*^D45E, D47E^*-f | ATTTTGGTGGATTTTGAAGAG |
| *mntA*_CL1_*^D45E, D47E^*-r | TCCACCAAAATCTCAACCTCGCTGT |
| *hepT*_CL1_*^Y82F^*-f | TTTGGCGTTGACGTTCGAGTGCT |
| *hepT*_CL1_*^Y82F^*-r | TCAACGCCAAAGAACGCGTGGAT |
| **Primers for protein expression and purification** | |
| pET28b-*mntA*_CL1_-f | CTAGCCATGGGCGCAATGCTGACCCTTGAAGAAATTGAGAGCATTCTGACAG |
| pET28b-*mntA*_CL1_(His)-r | CCCGGATCCCTAGTGGTGGTGGTGGTGGTGCTTCCTAAGCTTCTCAAAT |
| pET21-*mntA*-f | TACTTCCAATCCAATGCC ATGCAACAACTAAATGAAAATA |
| pET21-*mntA*-r | TTATCCACTTCCAATGTTATCAAAGTGACTCCCCCCTAT |
| *MntA*-f | CCGGAATTCATGCAACAACTAAATGAAAATA |
| *HepT-flag-r* | CGCGGATCCTTACTTATCGTCGTCATCCTTGTAATCCTCTGCTTTTATCACATCA |
| **Primers for DNA sequencing** | |
| PHGE-f | CACCTCGCTAACGGATTCACC |
| PHGE-r | CCAATACGCAAACCGCCTC |
| pET28b-f (T7-f) | TAATACGACTCACTATAGGG |
| pET28b-r (T7-r) | TATGCTAGTTATTGCTCAG |

**Table S5.** The theory molecular of His-tagged HepT and MntA (MntA^G27A, S28T^) expressed from pET28b-based plasmids.

| Plasmid | HepT | MntA or MntA^G27A, S28T^ | note |
| --- | --- | --- | --- |
| pET28b*-mntA-hepT-His* | 16135.42 Da | 15623.69 Da |  |
| pET28b*-hepT-His* | 16192.47 Da | - |  |
| pET28b*-mntA*^G27A, S28T^*-hepT-His* | 16135.42 Da | 15651.75 | Have not detected |

A Gly was introduced to the N terminus of the first gene expressed from pET28b plasmid.

**Table S6.** Putative HEPN/MNT pairs Selected for experimental verification. The gene ID were extracted from IMG/M database.

| Organism | Phylum | MntA Gene ID | A Size (aa) | HepT Gene ID | T Size (aa) |
| --- | --- | --- | --- | --- | --- |
| *S. putrefaciens* W3-18-1 | proteobacteria | 640069191 | 132 | 640069191 | 142 |
| *B. merycicum* DSM6492 | Actinobacteria | 2662148889 | 155 | 2662148888 | 122 |
| *T. cleftensis* CL1 | Euryarchaeota | 2518820817 | 99 | 2518820818 | 110 |
| *M. mazei* TUC01 | Euryarchaeota | 2540563384 | 134 | 2540563385 | 140 |

The TA pair HepT/MntA_W3-18-1_ was wrong annotated to be a fusion protein.


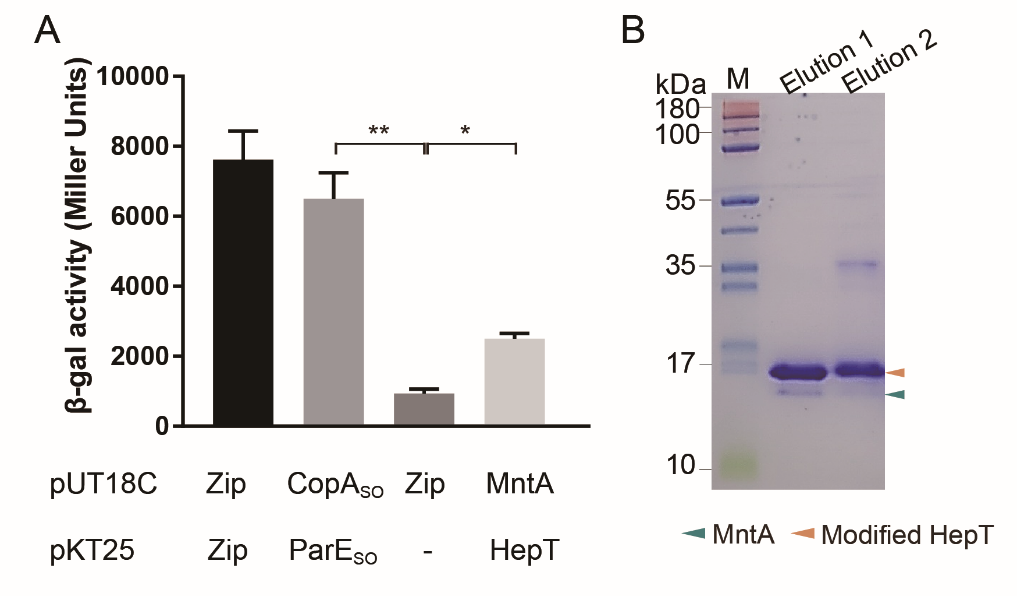


**Figure S1.** Purification of the modified toxin HepT from *E. coli* cell lysate co-expressing *hepT* and *mntA*. Tricine SDS-PAGE of purified modified HepT isolated from strain *E. coli* BL21/pET28b-*mntA-hepT-*His. The cell lysate was vigorous shaken before loading the resin for purification, the modified HepT (with purify > 90%) was obtained after second elution (Elution 2) from the TA complex by removing the MntA.


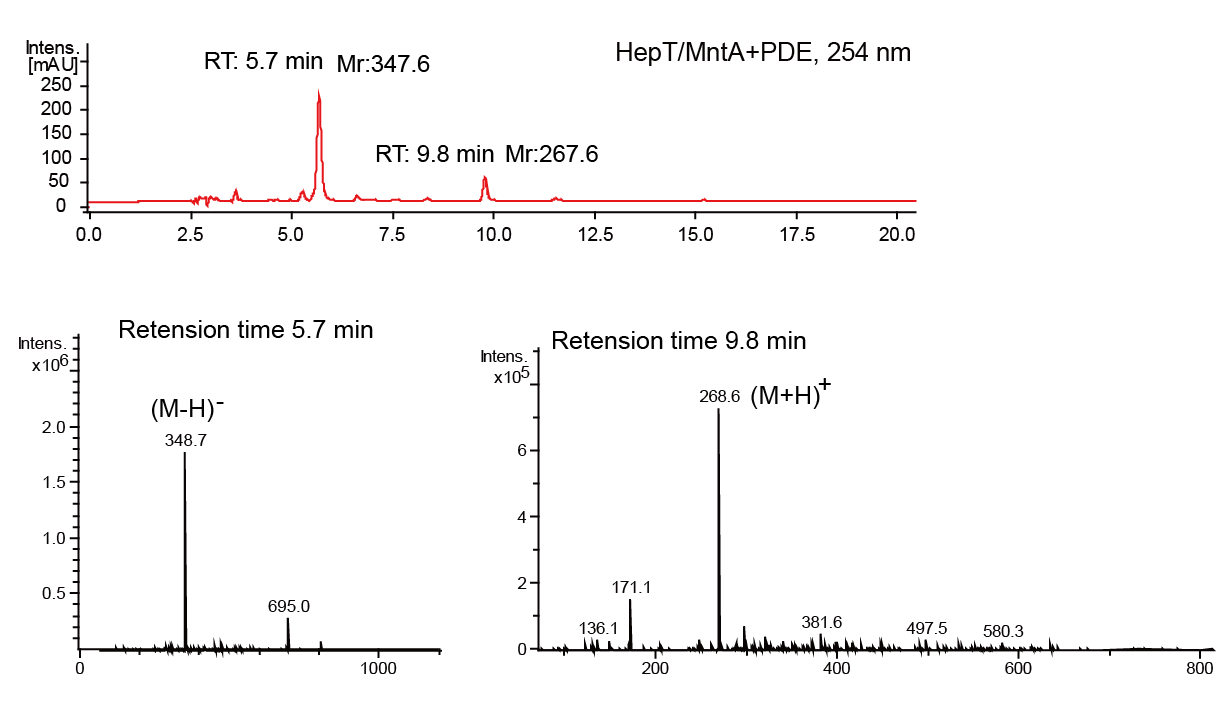


**Figure S2.** **LC-MS analysis of the PDE cleaved products of HepT/MntA complex**. LC profile of PDE cleaved products of HepT/MntA complex (up). ESI-MS analysis of LC fractions containing the 5.7 min product and 9.8 min product are shown (bottom).


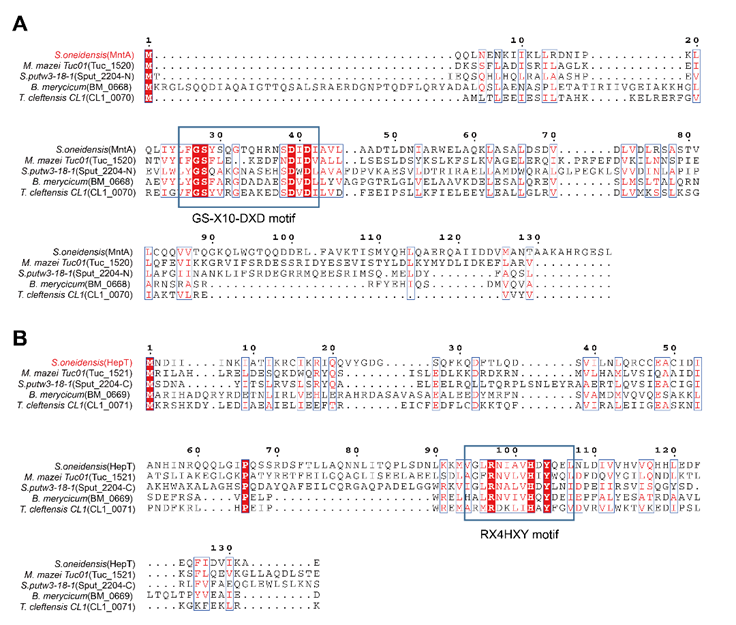


**Figure S3. Sequence alignment of MntA and HepT with its representative homologs from different strains**. Sequence alignment of MntA **(A)** and HepT **(B)** were performed by the MAFFT program online and enhanced by ESPript 3.0. The HEPN/MNT pairs including HepT/MntA from *S. oneidensis,* *Shewanella* sp. W3-18-1, *Bifidobacterium merycicum* DSM6492, archaeon *T. cleftensis* CL1 and *Methanosarcina mazei* TUC01. The conserved motif GSX_10_DXD in MntA proteins and the conserved motif RX_4_HXY in HepT proteins are boxed. All the sequences information was listed in **Table S4**.

**
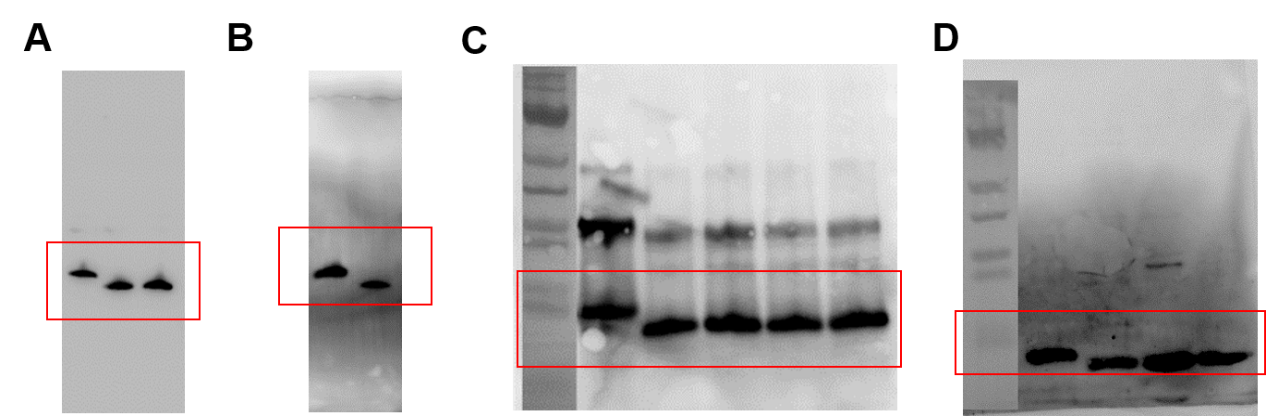
**

**Figure S4. Original western blot images.** **(A)** Gel from figure 2B. **(B)** Gel from figure 2C. **(C)** Gel from Figure 2E. **(D)** Gel from Figure 7B.

**
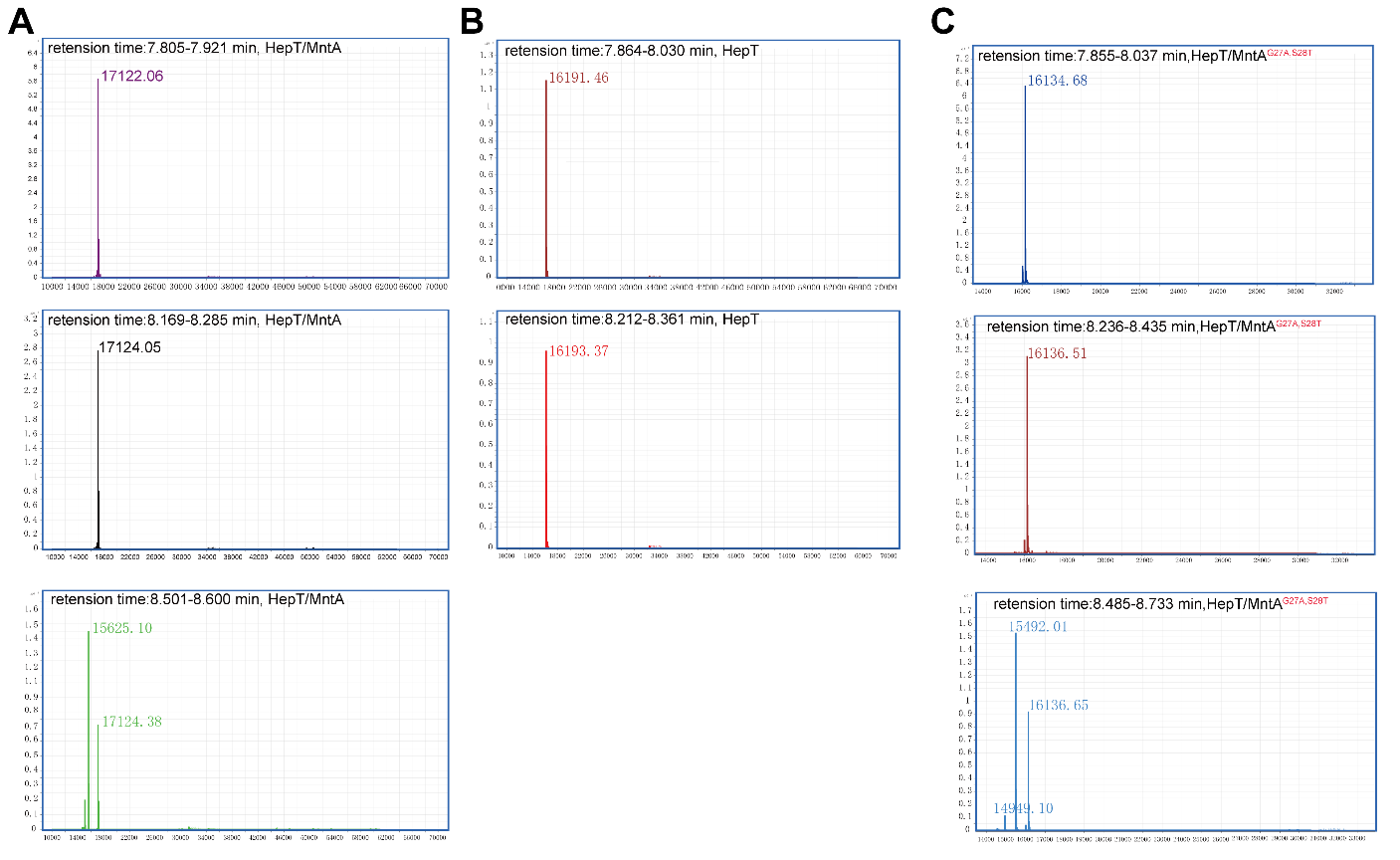
**

**Figure S5.** **Mass spectra of the peaks indicated in Figure 2F.** **(A)** Mass spectra of the peaks at the indicated retention time of HepT/MntA complex expressed from pET28b-*mntA-hepT*-Chis. **(B)** Mass spectra of the peaks at the indicated retention time of his-tagged HepT expressed from pET28b*-hepT*-Chis. **(C)** Mass spectra of the peaks at the indicated retention time of HepT/MntA^G27A, S28T^ complex expressed from pET28b-*mntA*^G27A, S28T^*-hepT*-Chis. Theoretical molecular weight of MntA and HepT were performed by ProtParam software online (<https://web.expasy.org/protparam/>) and listed on **Table S5**.


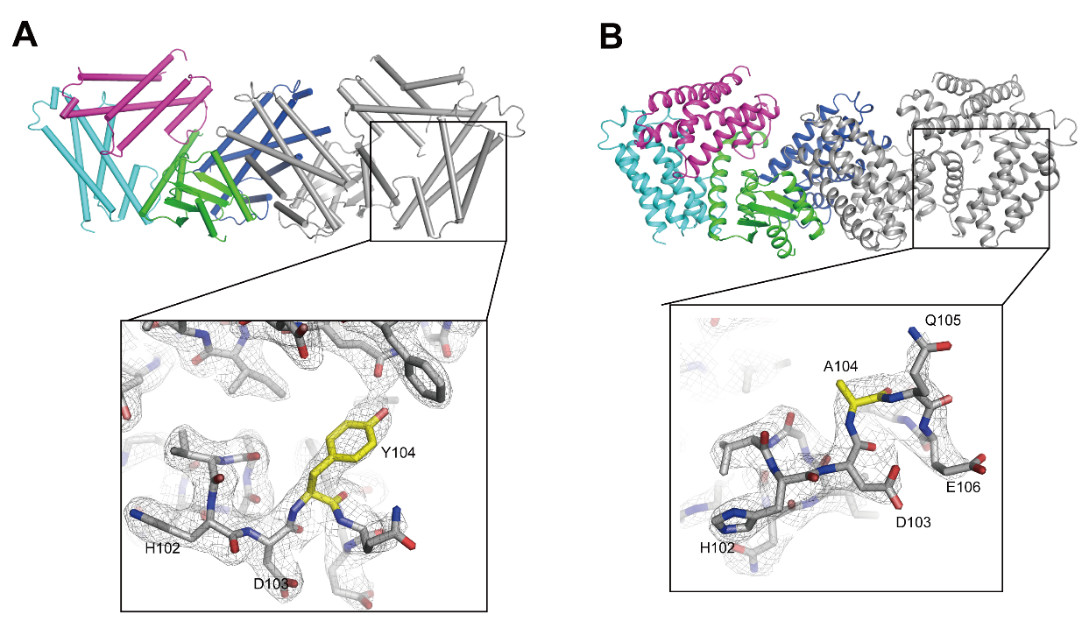


**Figure S6. Structure analysis of HepT^Y104A^/MntA and HepT/MntA^D39E, D41E^. (A)** Overall crystal structure of the HepT/MntA ^D39E, D41E^ complex shown as a cartoon. **(B)** Overall crystal structure of the HepT^Y104A^/MntA complex shown as a cartoon. HepT-MntA ^D39E, D41E^ and HepT^Y104A^/MntA were not AMPylated. The 2Fo-Fc omit map of Y104 of HepT and the local residues, which was contoured at 1.0 σ level.


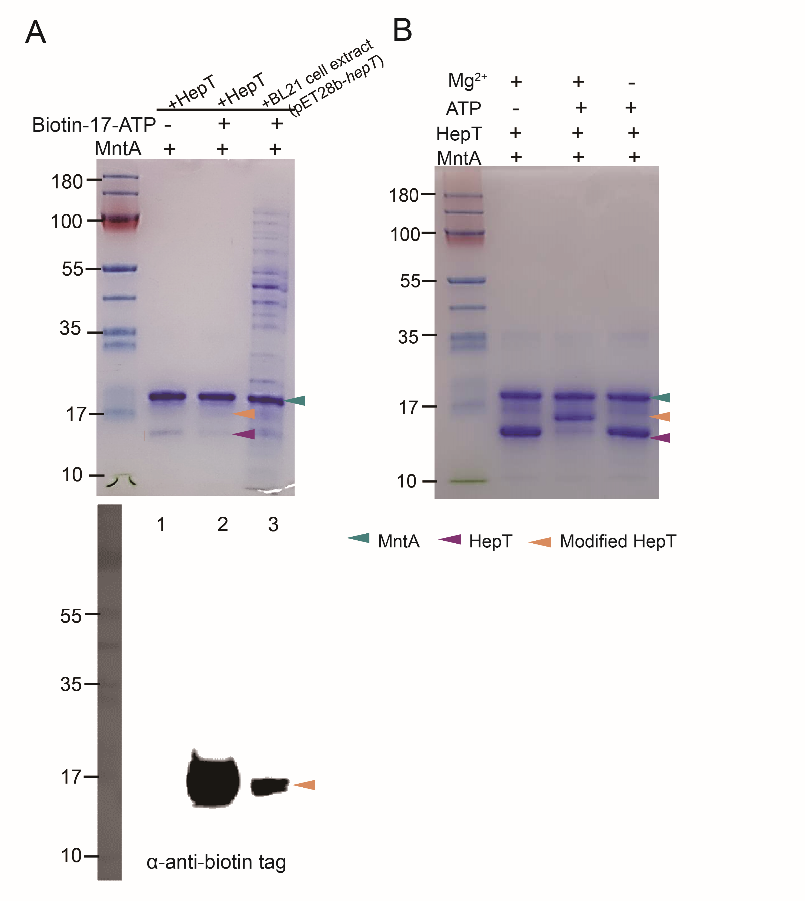


**Figure S7. (A) MntA mediated the transfer of AMPs to HepT toxin using ATP as the substrate.** Purified MntA was incubated with purified HepT or with *E. coli* cell lysate containing HepT with biotin-17-ATP as the substrate (upper panel), and a Western blot was performed using anti-biotin tag antibodies for the same set of samples (lower panel). **(B)** MntA modified HepT in the presence of Mg^2+^ ions but not in the absence of Mg^2+^ ions.


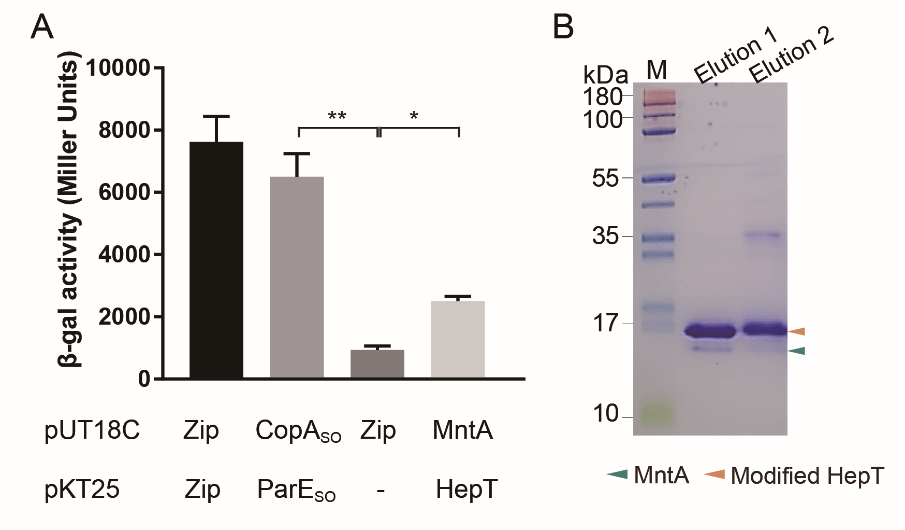


**Figure S8.** The quantitative analyses of the BACTH assay was performed as previously described (2). β-galactosidase assay was performed to assess the interactions between MntA and HepT. A type II TA pair ParE_SO_ and CopA_SO_ was used as the control (2).


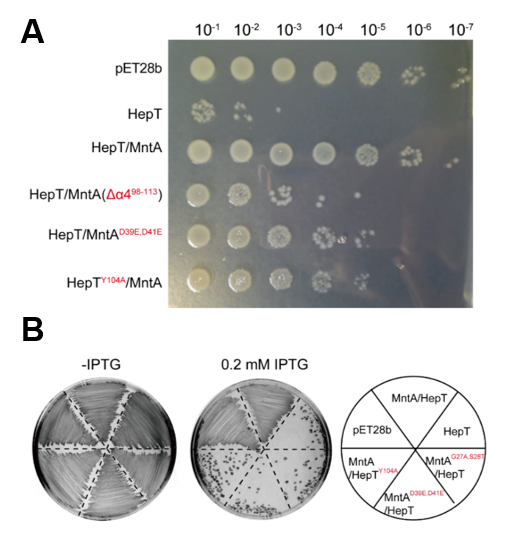


**Figure S9. AMPylation is critical for MntA to neutralize the toxicity of HepT. (A)** BL21 (DE3) hosts carrying the pET28b-based plasmids were cultured in LB medium with 50 μg/mL kanamycin, and 0.5 mM IPTG were added at a turbidity of 0.1 at 600 nm. The cells induced for 4 h were serially diluted, dropped onto LB plates and incubated at 37°C for 12 hr. **(B)** BL21 (DE3) hosts carrying the pET28b-based plasmids were streaked onto LB plates with 50 μg/mL kanamycin with or without 0.2 mM IPTG, and were incubated for 12 hr. Three independent cultures were evaluated for each; only one representative image is shown here.


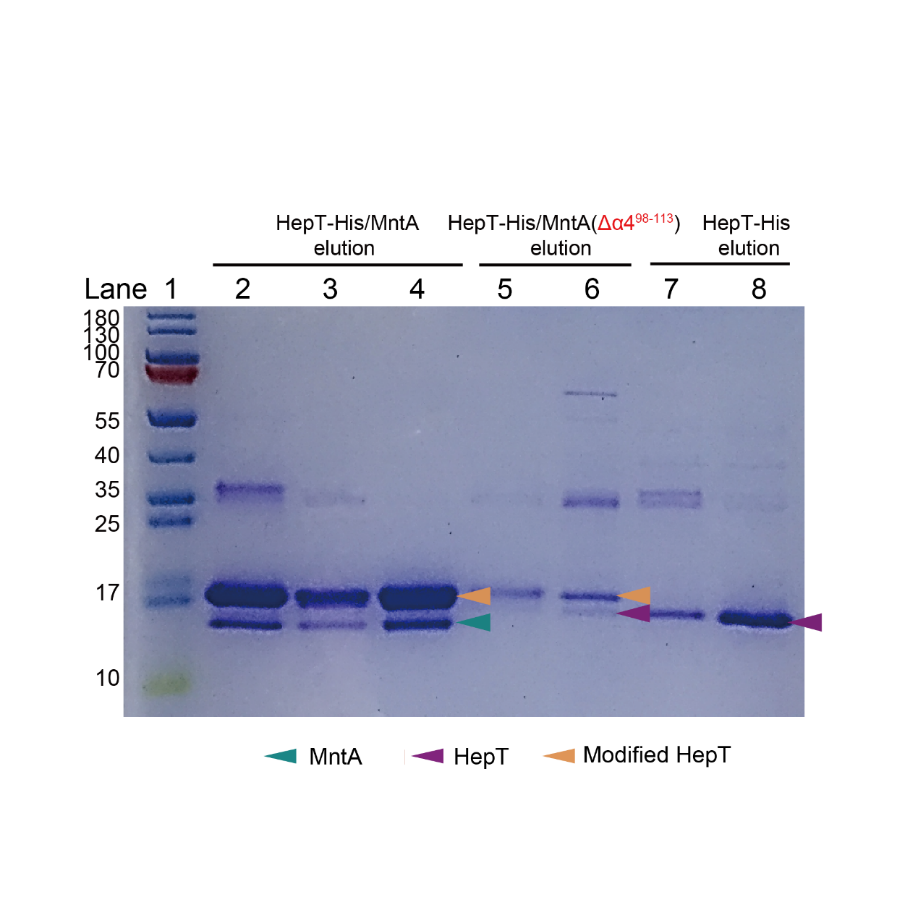


**Figure S10. Tricine** **SDS-PAGE of purified HepT/MntA, HepT/MntAΔα4^98-113^ and HepT.** HepT/MntA complex and its variants HepT/MntAΔα4^98-113^, sole HepT were purified from pET28b-based plasmid in *E. coli* BL21 (DE3). The Tricine SDS-PAGE reveals that antitoxin MntA lacks the α4^98-113^ cannot be pull-down by HepT and HepT were not fully AMPylated.

**
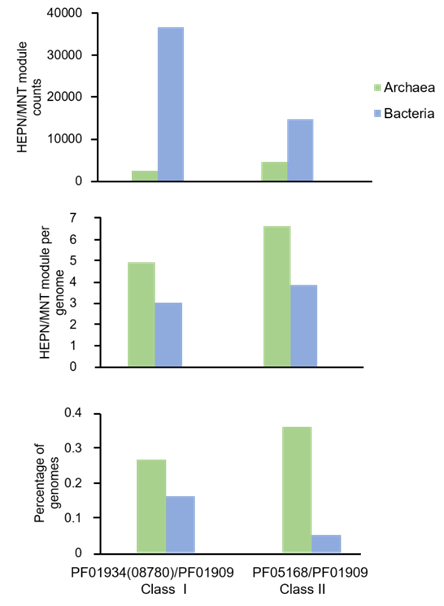
**

**Figure S11. Prevalence of HEPN/MNT pairs in bacteria and archaea.** The number of HEPN/MNT pairs **(up)**, the average number of HEPN/MNT pairs per genome **(middle)** and the percentage of genomes identified with HEPN/MNT pairs **(bottom)** of PFAM families containing HEPN/MNT pairs in bacteria and archaea. Data was retrieved from the IMG/M database based on one MNT-domain (PF01909, NTP_transf_2) and three HEPN homologous domains (PF01934, DUF86; PF08780, NTase_sub_bind; PF05168, HEPN).


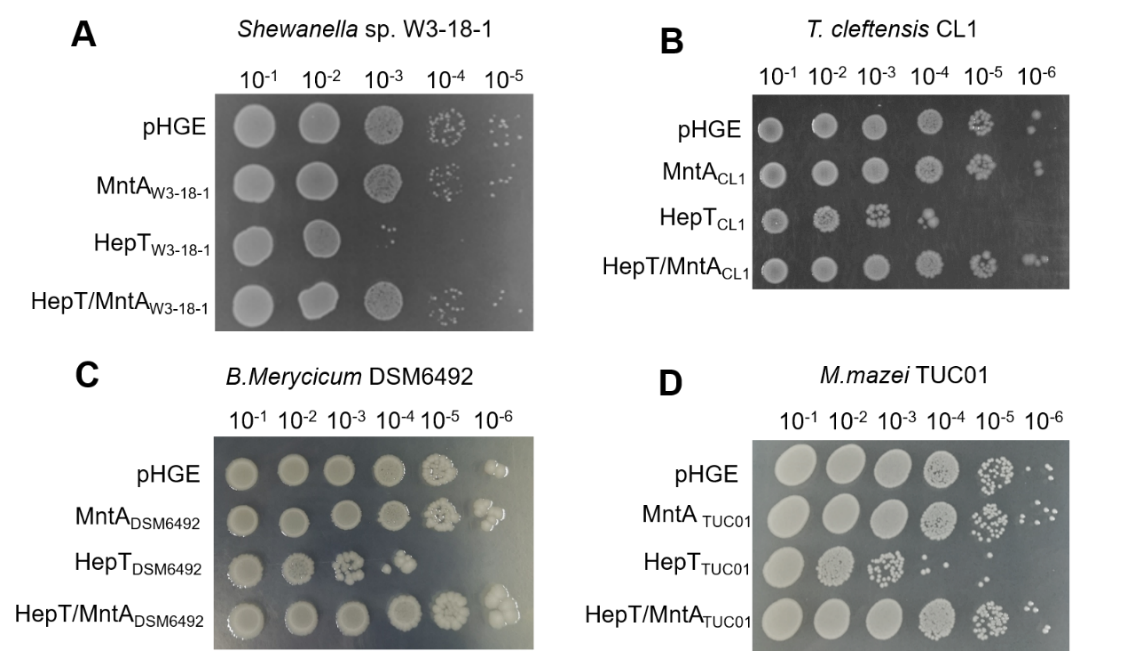


**Figure S12. HepT and MntA with the conserved signature motifs form a TA pair in various bacteria and archaea.** HEPN/MNT pairs from *Shewanella* sp. W3-18-1 **(A)**, *Bifidobacterium merycicum* DSM6492 **(B)** *T. cleftensis* CL1**(C)** and *Methanosarcina mazei* TUC01 **(D)** were cloned into plasmid pHGE. *E. coli* BW25113 carrying the pHGE-based plasmids were determined with 0.5 mM IPTG added at a turbidity of 0.1 at 600 nm. The cells induced for 2 h were serially diluted, dropped onto LB plates and incubated at 37°C for 16 hr. Three independent cultures were evaluated for each; only one representative image is shown here.


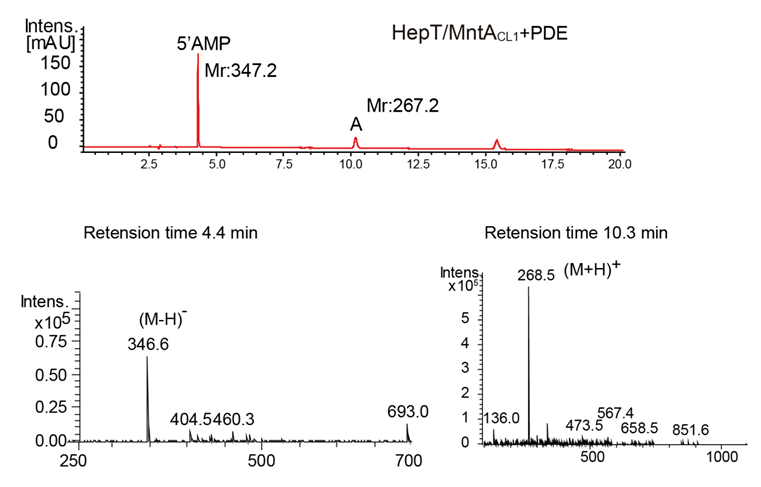


**Figure S13.** **LC-MS analysis of the PDE cleaved products of HepT/MntA_CL1_ complex**. LC profile of PDE cleaved products of HepT/MntA_CL1_ complex (up). ESI-MS analysis of LC fractions containing the 4.4 min product and 10.3 min product were shown (bottom).

**REFERENCES**

1. Baba, T., Ara, T., Hasegawa, M., Takai, Y., Okumura, Y., Baba, M., Datsenko, K.A., Tomita, M., Wanner, B.L. and Mori, H. (2006) Construction of *Escherichia coli* K-12 in-frame, single-gene knockout mutants: the Keio collection. *Mol. Syst. Biol.*, **2**, 2006.0008.

2. Yao, J., Guo, Y., Wang, P., Zeng, Z., Li, B., Tang, K., Liu, X. and Wang, X. (2018) Type II toxin/antitoxin system ParE_SO_/CopA_SO_ stabilizes prophage CP4So in *Shewanella oneidensis*. *Environ. Microbiol.*, **20**, 1224-1239.

3 Yao, J., Guo, Y., Zeng, Z., Liu, X., Shi, F. and Wang, X. (2015) Identification and characterization of a HEPN-MNT family type II toxin-antitoxin in *Shewanella oneidensis*. *Microb. Biotechnol.*, **8**, 961-973.
